# Rice Bran Arabinoxylan Compound and Tryptophan Metabolism on Quality of Life of Cancer Patients: A Secondary Analysis of the RBAC-QoL Study

**DOI:** 10.1177/11786469261441904

**Published:** 2026-04-30

**Authors:** Soo Liang Ooi, Benjamin Kimble, Benjamin S. Pak, Peter S. Micalos, Sok Cheon Pak

**Affiliations:** 1 School of Dentistry and Medical Sciences, Charles Sturt University, Bathurst, NSW, Australia; 2 Sydney School of Veterinary Science, The University of Sydney, NSW, Australia; 3 The Centre for Molecular Oncology, School of Biomedical Sciences, The University of New South Wales, Sydney, NSW, Australia; 4 School of Dentistry and Medical Sciences, Charles Sturt University, Port Macquarie, NSW, Australia

**Keywords:** biobran, L-tryptophan, L-kynurenine, blood markers, patient-reported outcome measures, QLQ-C30

## Abstract

Tryptophan metabolism, with its breakdown along the kynurenine pathway, is linked to the diminishing quality of life (QoL) of cancer patients. Rice bran arabinoxylan compound (RBAC) is a plant-based immunomodulator shown to improve the global QoL and functioning beyond the placebo during systemic cancer treatment in a randomised-controlled trial (RBAC-QoL study). However, whether RBAC improved QoL through the tryptophan pathway, and the role of tryptophan and its kynurenine metabolite in these QoL outcomes, were not previously investigated. In this study, serum tryptophan and kynurenine were analysed in samples collected from the RBAC-QoL study using a validated liquid chromatography method with ultraviolet and fluorescence detection. This secondary analysis was conducted using repeated-measures ANOVA, Spearman’s correlation, and linear mixed models. The results show that RBAC supplementation had no statistically significant effect on tryptophan metabolism compared to the placebo. Tryptophan significantly (*P* ⩽ .05) correlated positively with global QoL, physical and social functioning and negatively with fatigue, dyspnoea, appetite loss and diarrhoea. Kynurenine also demonstrated significant (*P* ⩽ .05), but weaker correlations with physical and social functioning (positively), as well as fatigue and dyspnoea (negatively), albeit weaker than tryptophan. The kynurenine-to-tryptophan ratio exhibited no significant correlations with QoL. Stepwise reduction of a linear mixed model of haematological, renal, liver, and immune markers revealed that tryptophan and gamma-glutamyl transferase are prominent predictors of QoL (*P* < .001). RBAC and serum tryptophan appeared to have an additive effect on QoL. Future research should investigate the combined impact of RBAC and tryptophan to assess any potential synergistic effects.

**Trial registration:** The RBAC-QoL study was prospectively registered on the Australian New Zealand Clinical Trials Registry (ANZCTR Reg No: ACTRN12619000562178p, 10/04/2019).

## Introduction

Cancer and its treatment impact the health-related quality of life (QoL) in many ways. Patients often experience a range of symptoms, including pain, fatigue, dyspnoea, loss of appetite, and others.^1^ Treatments, such as chemotherapy or radiation, often lead to unwanted side effects, including nausea, vomiting, constipation, alopecia and peripheral neuropathy, which could compromise daily functioning.^2^ This high symptom load is also associated with increased levels of emotional suffering, such as anxiety and depression, and poor physical and societal functioning, all of which impact patients’ QoL.^3^ Hence, the QoL outcome of patients is a crucial clinical consideration in cancer management.^4-6^ Complementary and supportive care alongside primary cancer treatments is needed to enhance overall well-being and improve disease outcomes.^7,8^

Rice bran arabinoxylan compound (RBAC) is a plant-based food supplement that has exhibited immune restorative function in cancer patients through upregulating natural killer cell activity and enhancing inflammatory and cytotoxic responses.^9^ Prior systematic reviews of available research evidence found RBAC to improve the QoL of cancer patients when used as a complementary therapy with conventional cancer treatment.^9,10^ A recent double-blind, placebo-controlled pilot trial (RBAC-QoL study) also demonstrated that RBAC improved global QoL scores as well as role and social functioning of cancer patients beyond placebo effects during systemic cancer treatment.^11^ Patients in the RBAC group also reported lower symptom scores in fatigue, pain, dyspnoea, and appetite loss compared to the placebo group. Although it was suggested that RBAC could improve QoL by enhancing nutritional status and modulating immune function, the biological pathway by which RBAC impacts perceived QoL remained unclear.^11^

Tryptophan is an essential amino acid needed for many human biological functions. As a precursor protein, tryptophan is synthesised into many bioactive compounds, including nicotinamide (vitamin B3), serotonin (neurotransmitter), melatonin (hormone), tryptamine, kynurenine, and many others with wide-ranging roles in human health.^12^ About 95% of overall tryptophan is degraded through the kynurenine metabolic pathway, whose first reaction is catalysed by tryptophan 2,3-dioxygenase (TDO) or indoleamine 2,3-dioxygenase 1 (IDO1).^13^ Diminished tryptophan levels due to chronic inflammation could lower immune responsiveness and influence mood, physical strength, and haematopoiesis, thus affecting the QoL.^14^ Specifically, in cancer patients, the inflammation-induced breakdown of tryptophan along the kynurenine pathway was found to relate to fatigue, depression, and anaemia.^15^ As such, tryptophan metabolism and signalling have garnered considerable attention in cancer research as therapeutic strategies for enhancing patient outcomes.^16^

The present research is a secondary analysis of the RBAC-QoL data, which aimed to explore the relationship between RBAC supplementation and tryptophan metabolism in cancer patients and validate whether the tryptophan-kynurenine pathway could be a determinant of QoL. The research questions are: (1) In cancer patients under active treatment, could RBAC supplementation affect the levels of tryptophan, kynurenine, and kynurenine to tryptophan ratio (KTR)? (2) Are tryptophan, kynurenine, and KTR related to the reported QoL outcomes of the patients? (3) Are tryptophan, kynurenine, and KTR significant factors in predicting QoL compared to other blood markers for haematological, immune, inflammatory and nutritional markers?

## Materials and Methods

The RBAC-QoL study was a randomised, placebo-controlled, pilot trial conducted from June 2020 to April 2024 in New South Wales, Australia. The specifics of the trial, including the study protocol,^17^ interim analysis,^18^ and the final report^11^ have been published for open access. Briefly, the trial recruited adult patients with any solid organ cancer (⩾ stage II) who were undergoing outpatient chemotherapy or immunotherapy. Participants were randomly assigned to consume either RBAC or a placebo powder as an oral supplement (3 g/day) for 24 weeks while continuing their oncological treatment. During the trial, the participants attended 5 study visits (6 weeks apart) to complete QoL questionnaires and underwent blood tests. The data collectors and the treating oncologists of the participants were blinded to group assignments. The RBAC-QoL study was approved by the Human Research Ethics Committee (HREC) of Concord Repatriation General Hospital, Sydney Local Health District (Application No. 2019/ETH00489) and the Charles Sturt University HREC (Protocol No. H19244). All participants provided written extended informed consent, allowing their data and biological samples to be used for future, related or extended projects. For more details, readers are encouraged to refer to the study publications.

### Participant Profile and Blood Markers

The following data from the RBAC-QoL study were used in the current analysis: (1) Participant profile including age, sex, cancer type, stage, and primary treatment, and their group assignment; (2) QoL outcome measures based on the European Organisation for the Research and Treatment of Cancer (EORTC) core 30-item QoL questionnaire (QLQ-C30); (3) Blood test results from routine pathological screening including the full blood count, liver function tests, electrolytes, urea, creatinine, C-reactive protein (CRP), and prealbumin; (4) 15 cytokine/chemokine markers, including granulocyte-macrophage colony-stimulating factor, interferon-gamma (IFN-γ), interleukin (IL)-1β, IL-1RA, IL-2, IL-4, IL-5, IL-6, IL-8, IL-10, IL-12p40, IL-12p70, IL-13, monocyte chemoattractant protein-1, and tumour necrosis factor-alpha (TNF-α). The cytokine/chemokine markers were analysed using multiplex quantification via Luminex xMAP technology (Luminex, Austin, TX, USA), conducted by Eve Technologies Corp. (Calgary, AB, Canada). The methodology for sample collection, storage, and cytokine/chemokine marker assay has been reported previously.^17,18^

### QoL Measures

QoL measurement was based on the scoring of QLQ-C30, following the procedures stipulated by EORTC.^19^ This study included the global QoL scale, 5 functional scales (physical, role, emotional, cognitive, and social), 9 symptom-related measures (fatigue, nausea and vomiting, pain, dyspnoea, insomnia, appetite loss, constipation, diarrhoea) and a single financial impact score for analysis. The summary score (SQ), calculated as the mean of the combined 13 QLQ-C30 scale and item scores (excluding the global QoL and financial impact items), was used as the primary indicator of a patient’s overall QoL. SQ is a more reliable and robust QoL measure in oncology research compared to individual scale and item scores.^20^

### Tryptophan and Kynurenine Assay

Levels of tryptophan and kynurenine (Sigma Aldrich, Castle Hill, NSW, Australia) were analysed using non-fasting blood samples prepared into serum collected from the RBAC-QoL study, applying a previously validated liquid chromatography (LC) method^21^ with minor modifications. The LC system consisted of a Shimadzu Nexera XR LC system (Rydalmere, NSW, Australia) with these binary mobile phases: 20 mM ammonium acetate (adjusted to pH 4.5 with acetic acid) mixed with either 5% acetonitrile (A) or 70% acetonitrile (B). The following gradient was applied at a flow rate of 1.2 ml/min: 0 to 1 minute (0%-5% B), 1 to 2 minutes (5% B), 2 to 5 minutes (5%-50% B), 5 to 10 minutes (50%-90% B), 10 to 11 minutes (90% B), and 11 to 12 minutes (90%-0% B). The stationary phase was a Waters X-Bridge C18 column (3.5 µm, 4.6 mm × 150 mm; Dundas, NSW, Australia), with the column temperature maintained at 35°C.

Tryptophan and kynurenine were monitored using fluorescence detection (excitation: 297 nm; emission: 347 nm) and ultraviolet detection (360 nm), respectively. Serum samples (50 µl) underwent protein precipitation with the addition of 100 µl methanol. The mixture was vortexed and centrifuged at 14 000×*g* for 10 minutes. The resulting supernatant was transferred to the injection vial, and 10 µl was injected into the HPLC system. The total run time was 15 minutes, with tryptophan and kynurenine eluting at 3.95 and 2.8 minutes, respectively. Quantification was performed using external calibration curves with quantification ranges of 1.91 µM (limit of quantification [LOQ]) to 122.41 µM for tryptophan and 0.94 µM (LOQ) to 60.03 µM for kynurenine. The precisions and accuracies at LOQ levels were within acceptable ranges for both tryptophan (precision: 1.35% coefficient of variation [CV], n = 3; accuracy: <15.41% of nominal concentration) and kynurenine (precision: 4.82% CV, n = 3; accuracy: <18.43%), following The International Council for Harmonisation of Technical Requirements for Pharmaceuticals for Human Use (ICH).^22^

### Statistical Methods

Statistical analysis was performed using RStudio version 2024.12.1 Build 563 (Posit, Boston, MA, USA), running R version 4.4.2. Every data point collected from participants in the trial was used regardless of withdrawal (intention-to-treat principle). Repeated measures analysis of variance (RM-ANOVA) was used to compare between-group differences over multiple time points, with F statistics, degrees of freedom, *P*-values, and effect sizes reported after sphericity correction. Pairwise comparisons were conducted when statistical significance was observed with the false discovery rates (fdr) applied to adjust the *P*-values for multiple comparisons. If any significant between-group differences in the baseline characteristics were identified, an analysis of covariance (ANCOVA) was to be performed on the significant outcomes from the primary analysis to account for potential confounding factors.

Spearman’s rank correlation (*r_s_*) was used to determine the strength and direction of the relationship between any 2 variables. The strength of the correlation coefficient was interpreted as negligible (*r*_s_ < .2), weak (.2 ⩽ *r*_s_ < .4), moderate (.4 ⩽ *r*_s_ < .6), strong (.6 ⩽ *r*_s_ < .8), and very strong (*r*_s_ ⩾ .8). Similarly, the *P*-values of the correlation coefficients were adjusted for multiplicity with the fdr method. A linear mixed model was used to fit and analyse the significance of the relationship between the multiple factors that could determine an outcome variable. A stepwise backward reduction approach based on the Akaike information criterion was performed to reduce the fixed and random effects for the mixed model.

Continuous variables were reported as mean ± standard deviation. The difference between any 2 means was analysed using 2-sided Student’s *t*-statistics. Fisher’s exact test was used to determine if there were non-random associations between 2 categorical variables. Missing data were handled with pairwise deletion. A *P*-value of less than or equal to .05 was considered statistically significant.

## Results

### Participant Characteristics

Nineteen of the 29 participants in the RBAC-QoL study provided additional serum samples for further analysis. Hence, tryptophan and kynurenine assays were performed on this subgroup, and their baseline characteristics are shown in Table 1.

**Table 1.** Baseline Characteristics of the Participants.

N (available for analysis)		All	RBAC	Placebo	*P*-value
		19 (100%)	9 (47.4%)	10 (52.6%)	
Age		68.4 ± 7.03	71.9 ± 7.83	65.2 ± 4.56	*P* ** = .043** *
Sex	Male	16 (84.2%)	7 (77.8%)	9 (90.0%)	*P* = .582
	Female	3 (15.8%)	2 (22.2%)	1 (10.0%)	
Primary cancer	Melanoma	6 (31.6%)	4 (44.4%)	2 (20.0%)	*P* = .393
	Colon and rectum	5 (26.3%)	1 (11.1%)	4 (40.0%)	
	Lung	4 (21.0%)	2 (22.2%)	2 (20.0%)	
	Bladder	1 (5.3%)	1 (11.1%)	-	
	Oesophagus	1 (5.3%)	-	1 (10.0%)	
	Kidney	1 (5.3%)	-	1 (10.0%)	
	Stomach	1 (5.3%)	1 (11.1%)	-	
*Cancer stage*	III	5 (26.3%)	2 (22.2%)	3 (30.0%)	*P* = 1.0
	IV	14 (73.7%)	7 (77.8%)	7 (70.0%)	
*Recurrence*	No	11 (57.9&)	5 (55.6%)	6 (60.0%)	*P* = 1.0
	Yes	8 (42.1%)	4 (44.4%)	4 (40.0%)	
*Treatment*	Chemotherapy	12 (63.2%)	5 (55.6%)	7 (70.0%)	*P* = .650
	Immunotherapy	7 (36.8%)	4 (44.4%)	3 (30.0%)	
*Australian recommended food score*		32.5 ± 7.68	32.8 ± 7.60	32.2 ± 8.19	*P* *=* .870

All continuous variables are presented as mean ± standard deviation, and the hypothesis testing of 2 means was based on the 2-sided Student’s *t*-test. Statistical testing of categorical variables was determined using Fisher’s exact test.

* Significance: adjusted *P*-value ≤.05.

The participants were mainly older adults with later-stage cancer (stage III and IV), predominantly male (84.2%), with a mean age of 68.4 ± 7.03. Primary cancer sites were mainly skin (melanoma, 31.6%), colorectal (26.3%), and lung (21.0%), with a single count of bladder, oesophagus, kidney, and stomach cancer each. Slightly less than half (42.1%) had recurrent cancer. The participants were treated with either chemotherapy (63.2%) or immunotherapy (36.8%). Ten patients were from the placebo group, and 9 were from the RBAC group. There were no significant differences between groups in all characteristics except for age. The mean age of the RBAC group was significantly higher than that of the placebo group (71.9 ± 7.83 vs 65.2 ± 4.56, *P* = .043). Participants also completed the Australian Eating Survey Food Frequency Questionnaire to assess dietary quality.^23^ The mean Australian Recommended Food Scores of both groups were also not significantly different.

### Between-Group Analysis

The levels of tryptophan, kynurenine, and KTR were analysed with RM-ANOVA to determine the differences between groups. No significant difference was detected for tryptophan and kynurenine. However, analysis of KTR detected a marginally significant difference over time (F[4,48] = 2.864, *P* = .052, eta^2^[g] = 0.074) but not between the groups or the interaction of group and time. Pairwise analysis revealed no significant differences, which may be attributed to the small effect size.

**Figure 1.**
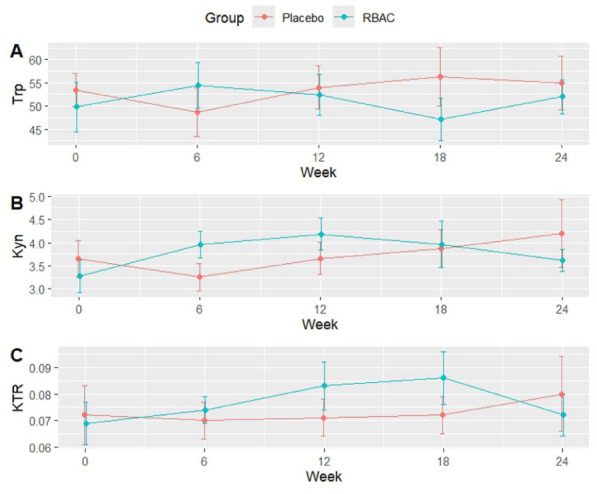
Plots of (A) tryptophan (Trp) at µM unit, (B) kynurenine (Kyn) µM unit, and (C) kynurenine (µM) to tryptophan (µM) ratio (KTR) for RBAC and placebo groups over time. Abbreviation: RBAC, rice bran arabinoxylan compound.

Figure 1 shows the between-group differences of tryptophan, kynurenine, and KTR over time. The levels of tryptophan, kynurenine, and KTR fluctuated, and no clear trend was detected. Adjusted analysis was performed with age as a covariate to account for the significant between-group differences in age. The results of the ANCOVA analysis revealed no significant differences between groups for all 3 parameters. The details of the data analysis are presented in the Supplemental Material.

### Tryptophan and QoL Outcomes

**Figure 2.**
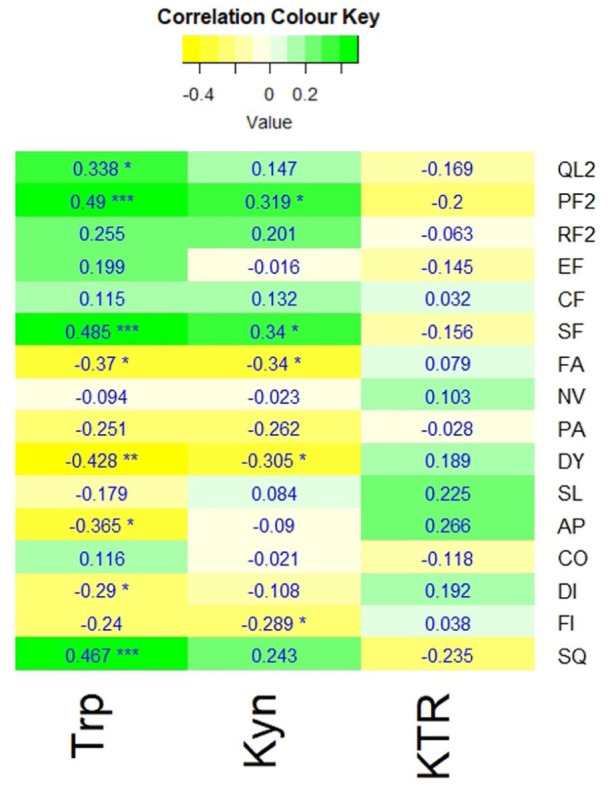
A heatmap showing the Spearman’s correlation coefficients of tryptophan (Trp), kynurenine (Kyn), and kynurenine to tryptophan ratio (KTR) with the QLQ-C30 measures: global QoL scale (QL2), physical (PF2), role (RF2), emotional (EF), cognitive (CF), and social (SF) functions, as well as fatigue (FA), nausea & vomiting (NV), pain (PA), dyspnoea (DY), insomnia (SL), appetite loss (AP), constipation (CO), diarrhoea (DI), financial impact (FI) and the summary score (SQ). Significance: adjusted *P*-value ⩽ .05*, *P*-value ⩽ .01**, *P*-value ⩽ .001***.

Figure 2 shows the Spearman’s coefficients of tryptophan, kynurenine, and KTR with the QoL outcome measures based on the QLQ-C30.

Tryptophan levels of the participants showed significant positive correlations with global QoL (*r*_s_ = 0.338, *P* ⩽ .05), physical functioning (*r*_s_ = 0.49, *P* ⩽ .001), social functioning (*r*_s_ = 0.485, *P* ⩽ .001), and overall QoL measured with SQ, the summary score of QLQ-C30 (*r*_s_ = 0.467, *P* ⩽ .001). Significant negative correlations were also detected between tryptophan and the symptom scores of fatigue (*r*_s_ = −0.37, *P* ⩽ .05), dyspnoea (*r*_s_ = −0.428, *P* ⩽ .001), appetite loss (*r*_s_ = −0.365, *P* ⩽ .05), and diarrhoea (*r*_s_ = −0.29, *P* ⩽ .05).

Kynurenine also demonstrated significant positive correlations with physical (*r*_s_ = 0.319, *P* ⩽ .05) and social (*r*_s_ = 0.34, *P* ⩽ .05) functioning and negative correlations with fatigue (*r*_s_ = −0.34, *P* ⩽ .05) and dyspnoea (*r*_s_ = −0.305, *P* ⩽ .05); albeit weaker compared to those of tryptophan. Additionally, kynurenine levels were negatively correlated with financial impact (*r*_s_ = −0.289, *P* ⩽ .05). In contrast, the KTR exhibited no significant correlations with any of the QoL outcome measures.

### Significant Factors Influencing QoL Outcomes

**Figure 3.**
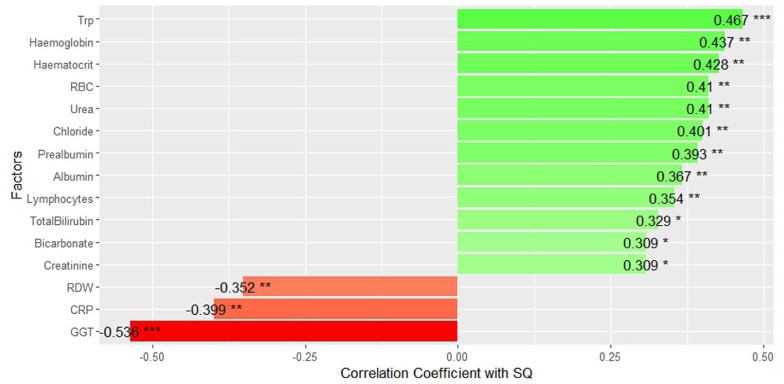
Significant factors correlate with the quality of life of the participants. Abbreviations: CRP, C-reactive protein; GGT, gamma-glutamyl transferase; RBC, red blood cell count; RDW, red cell distribution width; Trp, tryptophan. Significance: adjusted *P*-value ⩽ .05*, *P*-value ⩽ .01**, *P*-value ⩽ .001***.

Correlation analysis was conducted to compare all blood tests and cytokine markers with SQ, assessing how tryptophan and kynurenine, relative to other factors, influence QoL outcomes. Figure 3 illustrates the list of significant factors for SQ, arranged in order of decreasing correlation coefficients.

Many blood markers appeared to have a positive correlation with SQ, including haematological (red blood cell count [RBC], haemoglobin, haematocrit), electrolytes (chloride, bilirubin, bicarbonate), liver function (urea), and immune and nutritional markers (lymphocyte, albumin, prealbumin). Tryptophan was featured in the chart as the most prominent positive factor. Factors negatively correlated with SQ were the inflammatory marker, CRP, red cell distribution width (RDW) and gamma-glutamyl transferase (GGT). Serum cytokine levels, however, did not exhibit any significant correlations with SQ (see the Supplemental Material for the Spearman’s correlation test results for all factors).

### Linear Mixed Model Analysis

A linear mixed model, which accounted for group-specific effects and a random slope for weeks, was used to fit the data with SQ as the dependent outcome measure and all significant blood markers as fixed effects of the model. The model formula is shown below:

$$\begin{array}{l} SQ\sim Tryptophan+RBC+Haemoglobin\\ +Haematocrit+RDW+Lymphocytes\\ +Chloride+Bicarbonate+Urea+Creatinine\\ +TotalBilirubin+CRP+Prealbumin+Albumin\\ +GGT+(1+Week|Group) \end{array}$$

The details of the model fitting are provided in the Supplemental Material. Among the factors, only tryptophan (*P* < .001), GGT (*P* = .006), lymphocytes (*P* = .011), and total bilirubin (*P* = .045) were found to be significant predictors of SQ, with notable differences between groups.

Stepwise reduction of the full model above yielded a simplified model with only 2 independent predictors for the SQ score between groups after eliminating total bilirubin as the least significant predictor and the effect of weeks on the random slope. The simplified formula is shown below:

SQ~Tryptophan+GGT+(1|Group)

The fixed effect coefficient of tryptophan is 6.35 (*P* < .001) and GGT is −10,450.60 (*P* < .001) in this simplified model. The results of the model fitting are presented in the Supplemental Material.

**Figure 4.**
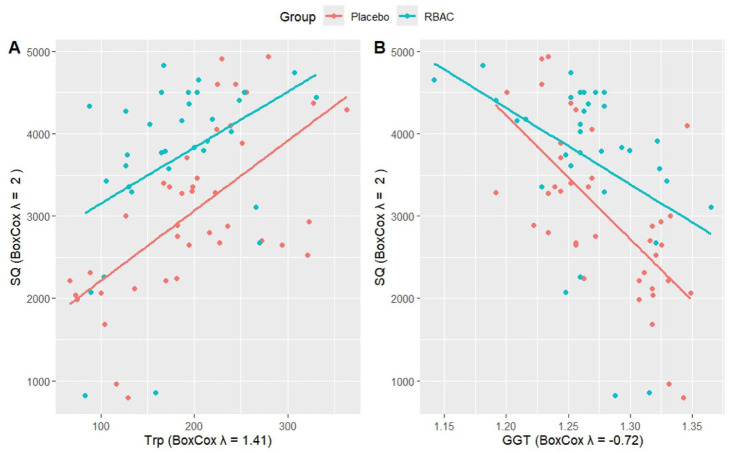
Visualisation of the linear model between the summary score for quality of life (SQ) and (A) tryptophan (Trp) and (B) gamma-glutamyl transferase (GGT) with group-specific effects after Box-Cox transformation.

Figure 4 presents a 2-dimensional visual representation of how tryptophan and GGT could predict SQ, comparing the RBAC and placebo groups. The increase in tryptophan was accompanied by the rise in SQ for both groups at a similar rate. Notwithstanding, the RBAC group had a consistently higher SQ than the placebo group at any level of tryptophan. Hence, there appeared to be an additive effect on SQ with RBAC and serum tryptophan level.

Higher GGT levels were associated with lower SQ, with both the RBAC and placebo groups exhibiting similar SQ levels at low GGT levels. However, the rate of deterioration between groups differed, with the drop in SQ being much steeper in the placebo group. Hence, in the case of liver function decline, indicated by elevated serum GGT, RBAC was more effective than placebo in preserving SQ.

## Discussion

This study explored the potential relationship between RBAC supplementation, tryptophan, and the associated kynurenine metabolite in cancer patients’ QoL. Tryptophan revealed significant correlations with several components of QoL, including a positive correlation with global QoL, physical and social functioning and a negative correlation with fatigue, dyspnoea, appetite loss and diarrhoea (see Figure 2). Moreover, serum tryptophan levels correlated significantly with the SQ, the summary score for QoL (*P* < .001); however, the correlation between kynurenine and KTR with SQ was not significant.

Several blood markers were also found to correlate with SQ, including haematological, renal, liver, immune, and nutritional markers. RBC, haemoglobin, haematocrit, RDW, lymphocytes, chloride, bicarbonate, urea, creatinine, total bilirubin, CRP, prealbumin, albumin, and GGT exhibited significant correlations with SQ scores. However, from the linear mixed model analysis with stepwise reduction, tryptophan remained 1 of 2 prominent independent factors that could predict SQ, with the other being GGT, a liver function marker.

These findings are consistent with previous reports in the literature. In a study by Schroecksnadel, Fiegl, Prassl, Winkler, Denz and Fuchs,^24^ cancer patients were grouped into those with remitting, stable, and progressive disease based on disease status and tumour progression. Tryptophan metabolic changes and QoL were measured. The study found patients with progressive disease had significantly lower tryptophan levels than those with stable disease or in remission (*P* < .01), and the decrease in tryptophan concentration was associated with decreased QoL (*r*_s_ = 0.256, *P* < .01) and increased fatigue (*r*_s_ = −0.179; *P* < .05). An earlier study among colorectal cancer patients with liver metastases also reported that reduced serum tryptophan was significantly associated with scores of physical symptoms (*r* = −.51, *P* = .01) and sickness impact profile (*r* = −.42, *P* = .01) that undermined QoL.^25^ Similarly, serum tryptophan was found to be a significant independent predictor of both physical symptoms (*P* < .01) and sickness impact (*P* < .04) using stepwise regression analysis.^25^

In another study by Kurz, Fiegl, Holzner, Giesinger, Pircher, Weiss, Denz and Fuchs,^26^ enhanced tryptophan degradation along the kynurenine pathway was also found to correlate significantly with fatigue (*r*_s_ = 0.376, *P* = .007), anaemia (*r*_s_ = 0.409, *P* = .003), and overall QoL scores (*r*_s_ = 0.382, *P* = .006) of lung cancer patients measured with the FACT (Functional Assessment of Cancer Therapy) questionnaires. It is worth noting that none of the earlier studies used the QLQ-C30 as the QoL questionnaire. Hence, the results from the present study validated the earlier findings with an internationally accepted QLQ-C30 measuring instrument, confirming the observation that higher serum tryptophan levels in cancer patients could mean higher overall QoL.

In the earlier reported results of the RBAC-QoL study, RBAC supplementation was shown to improve the QoL of cancer patients beyond placebo.^11^ The present analysis found little evidence suggesting that RBAC supplementation directly affected tryptophan and kynurenine levels and their ratio (KTR) over time. Hence, the positive impact of RBAC on QoL was not likely to result from preventing tryptophan degradation. Nonetheless, RBAC supplementation appeared to exhibit QoL-enhancing effects on the patients at the same tryptophan level.

A recent study demonstrated that administering L-tryptophan as a dietary supplement at 3 g/day significantly increased serum tryptophan levels (*P* < .001) in prostate, breast, or uterine cervical cancer and decreased symptom scores for hot flushes, asthenia, and insomnia, thus leading to overall QoL improvement (*P* < .001).^27^ A systematic review of 11 randomised controlled trials also found L-tryptophan supplementation effective in improving the mood of healthy individuals.^28^ Therefore, future research should investigate the combined impact of RBAC and L-tryptophan supplementation to determine whether any synergistic effects exist.

The liver enzyme GGT appeared to work independently from tryptophan, with rising serum GGT counteracting the tryptophan effect and lowering QoL. Not surprisingly, GGT is an extracellular enzyme involved in glutathione catabolism, a key antioxidant system that helps prevent cellular oxidative stress. Elevated serum GGT is an indication of liver injury or oxidative stress and has also been associated with increased cancer risk and poorer prognosis.^29,30^ While no study thus far explores the direct relationship between serum GGT and QoL of cancer patients, the liver function and QoL of cancer patients are known to be highly correlated.^31^

The differing rates of degradation in QoL due to increasing GGT between RBAC and placebo groups in the current study, as shown in Figure 4B, could likely be due to the hepatoprotective effects of RBAC, as demonstrated in several experimental and clinical studies.^32-36^ Thus, another area for future research could be to investigate the effect of RBAC on improving the QoL of cancer patients through the hepatoprotective pathways by quantifying changes in liver function enzymes including GGT, TDO expression, and biomarkers of oxidative stress, such as malondialdehyde, lipid peroxidation, and protein carbonyl levels.

Furthermore, given the positive correlation between tryptophan levels and social and physical function, future studies should also consider measuring downstream biomarkers, such as NAD⁺ (nicotinamide adenine dinucleotide) biosynthesis (relevant to physical function) or serotonin production (relevant to mood), to elucidate the possible biochemical pathways underlying better QoL. Another area of interest will be the potential effects of RBAC on systemic inflammation and its impact on tryptophan, kynurenine, and KTR, as the degradation of tryptophan along the kynurenine pathway is catalysed by IDO1, which is induced by inflammation.

The current study was a secondary analysis of existing data. While the results offered insights into how RBAC supplementation and serum tryptophan could affect the QoL of cancer patients, given its retrospective design, the study had inherent limitations. The study design of a parallel trial with repeated measures was not ideal for correlation analysis and predictive modelling. Hence, the observations derived in this study should not be used to infer causality in any way.^37^

In addition, the use of fasting blood samples for analysing tryptophan and its metabolites is ideal, as meal consumption can affect these biomarkers.^38^ This study was limited by the original study design and lacked access to fasting blood samples; future investigations should incorporate a more rigorous collection methodology for fasting blood samples.

IDO1 is an immunoregulatory enzyme whose activity could vary with age, sex, cancer type, cancer stage, metastatic burden and location, as well as treatment regimen (eg, chemotherapy or immunotherapy) in cancer patients.^39,40^ Hence, the small, heterogeneous sample size available for analysis in this study could not only reduce statistical power but also yield unreliable results.^41^ Thus, the present exploratory results should not be generalisable to the broader population of cancer patients.

Despite the limitations, this secondary analysis on RBAC and tryptophan could provide supporting data to inform future research, and the potential synergistic effects between RBAC and tryptophan warrant further investigation.

## Conclusion

This secondary analysis of the RBAC-QoL study found that RBAC supplementation did not affect the tryptophan metabolism among the participants. However, serum tryptophan levels demonstrated significant correlations with many aspects of QoL, including global QoL, physical and social functioning, and symptoms of fatigue, dyspnoea, appetite loss and diarrhoea. Furthermore, serum tryptophan was shown to be a prominent predictor of SQ, which is the summary score of QLQ-C30. RBAC supplementation and serum tryptophan appeared to have an additive effect on the QoL over placebo. Such findings provided supporting data to inform future research.

## Supplemental Material

sj-docx-1-try-10.1177_11786469261441904 – Supplemental material for Rice Bran Arabinoxylan Compound and Tryptophan Metabolism on Quality of Life of Cancer Patients: A Secondary Analysis of the RBAC-QoL Study Supplemental material, sj-docx-1-try-10.1177_11786469261441904 for Rice Bran Arabinoxylan Compound and Tryptophan Metabolism on Quality of Life of Cancer Patients: A Secondary Analysis of the RBAC-QoL Study by Soo Liang Ooi, Benjamin Kimble, Benjamin S. Pak, Peter S. Micalos and Sok Cheon Pak in International Journal of Tryptophan Research
