# Supplementary material for: Rice Bran Arabinoxylan Compound and Tryptophan Metabolism on Quality of Life of Cancer Patients: A Secondary Analysis of the RBAC-QoL Study
Source: Int J Tryptophan Res. 2026 Apr 30;19:11786469261441904. doi: 10.1177/11786469261441904 (PMC13150091; doi:10.1177/11786469261441904)
Supplement: sj-docx-1-try-10.1177_11786469261441904 – Supplemental material for Rice Bran Arabinoxylan Compound and Tryptophan Metabolism on Quality of Life of Cancer Patients: A Secondary Analysis of the RBAC-QoL Study [file sj-docx-1-try-10.1177_11786469261441904.docx]

Summary of Data Analysis for RBAC, Tryptophan, & QoL of Cancer Patients

Soo Liang Ooi^1^ Benjamin Kimble2 Benjamin S Pak^3^ Peter S Micalos^4^ Sok Cheon Pak^5^

2025-04-21

**library**(dplyr) **library**(readxl) **library**(knitr) **library**(forecast) **library**(rstatix) **library**(gplots) **library**(ggplot2) **library**(ggpubr) **library**(psych) **library**(tibble) **library**(lmerTest)

# Introduction

This R Markdown report is a supplementary document for the manuscript “Rice Bran Arabinoxylan Compound (RBAC) and Tryptophan Metabolism on Quality of Life (QoL) of Cancer Patients – A Secondary Analysis of the RBAC-QoL Study”. The following sections summarise the statistical analysis for the study.

# The Data

The following data is used in the analysis below:

1. Participant ID, age and group assignment;
2. QoL outcome measures based on the EORTC core 30-item QoL questionnaire (QLQ-C30);
3. Blood test results, including the complete blood count, liver and renal function tests, CRP, and prealbumin;
4. 15 cytokine/chemokine markers
5. Tryptophan, kynurenine and their ratio.

Outcome data measures (2 - 5) were collected from 5 study visits 6 weeks apart.

## Full data set

*# Let's load the data from the Excel file*

MyData <- read_excel("datafile.xlsx", sheet = "Data") DataDictionary <- read_excel("datafile.xlsx", sheet = "Dictionary") str(MyData)

## tibble [80 × 69] (S3: tbl_df/tbl/data.frame)

## $ row_id : num [1:80] 1 2 3 4 5 6 7 8 9 10 ...

## $ ID : num [1:80] 74 74 74 74 74 48 48 48 48 48 ...

## $ Group : chr [1:80] "Placebo" "Placebo" "Placebo" "Placebo" ...

| ## | $ | Week : | | num | [1:80] | 0 6 12 18 | | | | | 24 0 | 6 12 | | 18 24 ... | | | | |
| --- | --- | --- | --- | --- | --- | --- | --- | --- | --- | --- | --- | --- | --- | --- | --- | --- | --- | --- |
| ## | $ | Trp : | | num | [1:80] | 63.1 69.4 | | | | | 65.2 | 59.4 | | 60.4 ... | | | | |
| ## | $ | Kyn : | | num | [1:80] | 3.15 3.52 | | | | | 3.72 | 3.42 | | 3.7 ... | | | | |
| ## | $ KTR : | | | num | [1:80] | 0.0499 0.0508 0.0571 0.0576 0.0612 | | | | | | | | | | | ... | |
| ## | $ QL2 : | | | num | [1:80] | 75 83.3 83.3 66.7 83.3 ... | | | | | | | | | | |  | |
| ## | $ PF2 : | | | num | [1:80] | 100 100 100 100 100 ... | | | | | | | | | | |  | |
| ## | $ RF2 : | | | num | [1:80] | 100 100 100 100 100 ... | | | | | | | | | | |  | |
| ## | $ EF : | | | num | [1:80] | 91.7 91.7 83.3 91.7 100 ... | | | | | | | | | | |  | |
| ## | $ CF : | | | num | [1:80] | 100 100 100 100 100 ... | | | | | | | | | | |  | |
| ## | $ SF : | | | num | [1:80] | 100 100 100 100 100 ... | | | | | | | | | | |  | |
| ## | $ FA : | | | num | [1:80] | 11.1 0 0 11.1 11.1 ... | | | | | | | | | | |  | |
| ## | $ NV : | | | num | [1:80] | 0 0 0 0 0 ... | | | | | | | | | | |  | |
| ## | $ PA : | | | num | [1:80] | 33.3 0 16.7 0 0 ... | | | | | | | | | | |  | |
| ## | $ DY : | | | num | [1:80] | 0 0 0 0 0 ... | | | | | | | | | | |  | |
| ## | $ SL : | | | num | [1:80] | 0 0 33.3 33.3 0 ... | | | | | | | | | | |  | |
| ## | $ AP : | | | num | [1:80] | 0 | 0 | 0 | 0 | 0 | ... |  |  |  | | | | |
| ## | $ CO : | | | num | [1:80] | 0 | 0 | 0 | 0 | 0 | ... |  |  |  | | | | |
| ## | $ DI : | | | num | [1:80] | 0 | 0 | 0 | 0 | 0 | 0 0 | 0 | 0 0 | ... | | | | |
| ## | $ FI : | | | num | [1:80] | 0 | 0 | 0 | 0 | 0 | ... |  |  |  | | | | |
| ## | $ | SQ : | | num | [1:80] | 95.9 | | 99.4 | | | 94.9 | 95.9 | | 99.1 | ... | | | |
| ## | $ | AgeAtBaseline : | | num | [1:80] | 66.1 | | 66.1 | | | 66.1 | 66.1 | | 66.1 | ... | | | |
| ## | $ | Age : | | num | [1:80] | 66.1 | | 66.3 | | | 66.4 | 66.5 | | 66.6 | ... | | | |
| ## | $ | RBC | : | num | [1:80] | 4.2 4.2 4.1 4.3 4 4.5 4.4 4.3 4.4 4.5 ... | | | | | | | | | | | | |
| ## | $ | Haemoglobin | : | num | [1:80] | 135 135 129 135 129 139 134 135 137 139 ... | | | | | | | | | | | | |
| ## | $ | Haematocrit | : | num | [1:80] | 0.39 0.38 0.38 0.4 0.38 0.41 0.38 0.4 0.4 0.4 ... | | | | | | | | | | | | |
| ## | $ | MCV | : | num | [1:80] | 93 92 92 93 93 90 89 88 91 90 ... | | | | | | | | | | | | |
| ## | $ | MCH | : | num | [1:80] | 32 32 31.7 31.6 32 30.8 31.3 31.5 31.1 31.1 ... | | | | | | | | | | | | |
| ## | $ | MCHC | : | num | [1:80] | 347 351 344 341 344 343 352 358 343 344 ... | | | | | | | | | | | | |
| ## | $ | RDW | : | num | [1:80] | 13.8 12.8 13.1 12.7 12.5 12.3 13.2 13.1 13.4 13.2 ... | | | | | | | | | | | | |
| ## | $ | Platelet : | | num | [1:80] | 170 | | 177 | | 217 232 222 | | | | 219 210 | | 207 206 205 | | ... |
| ## | $ | WBC : | | num | [1:80] | 5.4 | | 5.7 | | 6.3 6.1 7.3 | | | | 6.7 8 7 | | 5.6 8.6 ... | |  |

## $ Neutrophils : num [1:80] 2.5 2.6 2.97 3.11 3.79 4.35 5.83 5.13 3.89 6.69 ...

## $ Lymphocytes : num [1:80] 2 2.3 2.44 2.2 2.49 1.76 1.56 1.36 1.24 1.27 ...

## $ Monocytes : num [1:80] 0.6 0.7 0.64 0.64 0.78 0.36 0.46 0.41 0.32 0.39 ...

## $ Eosinophils : num [1:80] 0.2 0.2 0.18 0.14 0.22 0.16 0.17 0.11 0.16 0.18 ...

## $ Basophils : num [1:80] 0 0 0.04 0.04 0.04 0.06 0.04 0.03 0.03 0.06 ...

140 144 144 144 143 143 ...

| ## | $ | Sodium : | num | [1:80] | 141 | 138 | 141 | 139 |
| --- | --- | --- | --- | --- | --- | --- | --- | --- |
| ## | $ | Potassium : | num | [1:80] | 4.2 | 4.3 | 4.7 | 4.9 |
| ## | $ | Chloride : | num | [1:80] | 107 | 106 | 107 | 107 |

5 4.3 4.2 4.2 4.4 4.1 ...

108 107 111 111 107 107 ...

## $ Bicarbonate : num [1:80] 28 28 26 25

| 26 29 25 25 | 27 26 ... |  |
| --- | --- | --- |
| 7.2 7.2 5.8 | 6.8 6.8 7.5 5.6 | ... |

## $ Urea : num [1:80] 7.8 8.4 8.7

## $ Creatinine : num [1:80] 92 106 110 95 100 75 70 70 83 100 ...

## $ TotalBilirubin: num [1:80] 7 6 7 7 5 13 9 9 11 11 ...

| ## | $ | CRP : | num | [1:80] | 0.4 | 0.9 | | 1.1 | 1.3 0.9 | | | 0.5 | 1.2 1.2 0.7 5.8 | | | ... |
| --- | --- | --- | --- | --- | --- | --- | --- | --- | --- | --- | --- | --- | --- | --- | --- | --- |
| ## | $ | Prealbumin : | num | [1:80] | 291 | 282 | | 265 | 296 270 | | | 375 | 335 335 274 276 | | | ... |
| ## | $ | Albumin : | num | [1:80] | 37 39 43 45 | | | | 44 50 45 45 42 | | | | | 49 | ... | |
| ## | $ | AGR : | num | [1:80] | 1 1.03 1.79 | | | | 1.88 1.76 ... | | | | |  |  | |
| ## | $ | AST : | num | [1:80] | 21 | 34 | 29 | 23 | 24 | 25 | 20 | 20 | 22 | 32 | ... | |
| ## | $ | ALT : | num | [1:80] | 35 | 39 | 35 | 31 | 35 | 30 | 25 | 25 | 30 | 34 | ... | |
| ## | $ | ALP : | num | [1:80] | 58 | 62 | 59 | 62 | 64 | 82 | 85 | 85 | 92 | 94 | ... | |
| ## | $ | GGT : | num | [1:80] | 20 | 21 | 16 | 20 | 20 | 23 | 26 | 26 | 15 | 19 | ... | |

| ## | $ | GMCSF : | | num | [1:80] | 270 391 194 411 520 ... | | |
| --- | --- | --- | --- | --- | --- | --- | --- | --- |
| ## | $ | IFNGamma : | | num | [1:80] | 9.8 12.3 8.8 12.5 11 7.5 11.5 8.5 7.8 17 ... | | |
| ## | $ | IL1Beta : | | num | [1:80] | 8 9.5 7 8 9.5 7.5 8.5 8.5 7.5 12 ... | | |
| ## | $ IL1RA : | | | num | [1:80] | 85 56.8 48.3 54 65.5 | 25.8 | 46 35.3 24.5 61.5 ... |
| ## | $ IL2 : | | | num | [1:80] | 20.3 18.5 15.5 21 21 | 18.3 | 20.3 18.5 14.5 25 ... |
| ## | $ | IL4 | : | num | [1:80] | 18.5 16.5 16 20 16.3 13 12.8 11 11.3 12 ... | | |
| ## | $ | IL5 | : | num | [1:80] | 46 36.5 35.5 38.3 47.8 18 20.5 16.5 15.3 46.5 ... | | |
| ## | $ | IL6 | : | num | [1:80] | 71.5 73.5 61.5 75 89.3 8 13.3 8.5 8.5 25 ... | | |
| ## | $ | IL8 | : | num | [1:80] | 203 249 184 292 268 ... | | |
| ## | $ | IL10 | : | num | [1:80] | 30.8 41.3 38.3 37.5 63.5 32 48 31.3 24.3 71.3 ... | | |
| ## | $ | IL12p40 | : | num | [1:80] | 21.3 21.5 17.8 21.5 27.3 13.5 13 12.5 13.5 21.8 ... | | |
| ## | $ | IL12p70 | : | num | [1:80] | 8.8 8 9.5 9.8 10.5 9.5 10 9.3 8.3 10.5 ... | | |
| ## | $ | IL13 | : | num | [1:80] | 13.3 13 11.5 11.5 14.5 12 12.5 10.5 12.3 17 ... | | |
| ## | $ | MCP1 | : | num | [1:80] | 8009 6998 7850 7725 7516 ... | | |
| ## | $ | TNFAlpha | : | num | [1:80] | 88 68.3 60 74.3 95 15.5 18.3 14 11 26.3 ... | | |

## Data dictionary

A data dictionary for the full dataset is displayed below:

kable(DataDictionary)

| **No** | **ColumnName** | **Description** | **Category** | **VariableType** | **UnitRange** |
| --- | --- | --- | --- | --- | --- |
| 1 | row_id | Sequential row index | NA | ordinal | (1 -  MaxRow) |
| 2 | ID | Unique Participant Indentifier | Participant | categorical | (1 - 90) |
| 3 | Group | Random group assignment | Participant | categorical | (RBAC,  Placebo) |
| 4 | Week | Number of weeks from baseline | Time | ordinal | (0, 6, 12, 18,  24) |
| 5 | Trp | Serum tryptophan level | Tryptophan- kynurenine | continuous | μM |
| 6 | Kyn | Serum kynurenine level | Tryptophan- kynurenine | continuous | μM |
| 7 | KTR | Kynurenine to tryptophan ratio | Tryptophan- kynurenine | continuous | (0 - 1) |
| 8 | QL2 | Global QoL score | QLQ-C30 | continuous | (0 - 100) |
| 9 | PF2 | Physical functioning | QLQ-C30 | continuous | (0 - 100) |
| 10 | RF2 | Role functioning | QLQ-C30 | continuous | (0 - 100) |
| 11 | EF | Emotional functioning | QLQ-C30 | continuous | (0 - 100) |
| 12 | CF | Cognitive functioning | QLQ-C30 | continuous | (0 - 100) |
| 13 | SF | Social functioning | QLQ-C30 | continuous | (0 - 100) |
| 14 | FA | Fatigue | QLQ-C30 | continuous | (0 - 100) |
| 15 | NV | Nausea and vomiting | QLQ-C30 | continuous | (0 - 100) |

| 16 | PA | Pain | QLQ-C30 | continuous | (0 - 100) |
| --- | --- | --- | --- | --- | --- |
| 17 | DY | Dyspnoea | QLQ-C30 | continuous | (0 - 100) |
| 18 | SL | Insomnia | QLQ-C30 | continuous | (0 - 100) |
| 19 | AP | Appetite | QLQ-C30 | continuous | (0 - 100) |
| 20 | CO | Constipation | QLQ-C30 | continuous | (0 - 100) |
| 21 | DI | Diarrhoea | QLQ-C30 | continuous | (0 - 100) |
| 22 | FI | Financial impact | QLQ-C30 | continuous | (0 - 100) |
| 23 | SQ | Summary of QoL scores | QLQ-C30 | continuous | (0 - 100) |
| 24 | AgeAtBaseline | The age of the participant at basline | Participant | continuous | (0 - 100) |
| 25 | Age | Age of the participant at each specific time point | Participant | continuous | (0 - 100) |
| 26 | RBC | Red blood cell count | Haematological | continuous | x10^12/L |
| 27 | Haemoglobin | Amount of haemoglobin in blood | Haematological | continuous | g/L |
| 28 | Haematocrit | The ratio of red blood cells to the total blood volume | Haematological | continuous | % |
| 29 | MCV | Mean cell volume | Haematological | continuous | fml |
| 30 | MCH | Mean corpuscular hemoglobin | Haematological | continuous | pg |
| 31 | MCHC | Mean corpuscular hemoglobin concentration | Haematological | continuous | g/L |
| 32 | RDW | Red cell distribution width | Haematological | continuous | % |
| 33 | Platelet | Platelet count in blood | Haematological | continuous | x10^9/L |
| 34 | WBC | White blood cell count | Haematological | continuous | x10^9/L |
| 35 | Neutrophils | Neutrophil count | Haematological | continuous | x10^9/L |
| 36 | Lymphocytes | Lymphocyte count | Haematological | continuous | x10^9/L |
| 37 | Monocytes | Monocyte count | Haematological | continuous | x10^9/L |
| 38 | Eosinophils | Eosinophil count | Haematological | continuous | x10^9/L |
| 39 | Basophils | Basophil count | Haematological | continuous | x10^9/L |
| 40 | Sodium | Sodium electrolyte test | Renal | continuous | mmol/L |
| 41 | Potassium | Potassium electrolyte test | Renal | continuous | mmol/L |
| 42 | Chloride | Chloride electrolyte test | Renal | continuous | mmol/L |
| 43 | Bicarbonate | Bicarbonate electrolyte test | Renal | continuous | mmol/L |
| 44 | Urea | Blood urea nitrogen (BUN) test | Renal | continuous | mmol/L |
| 45 | Creatinine | Serum creatinine levels | Renal | continuous | µmol/L |

| 46 | TotalBilirubin | Serum bilirubin levels | Liver | continuous | µmol/L |
| --- | --- | --- | --- | --- | --- |
| 47 | CRP | C-reactive protein (Inflammatory proteins) | Others | continuous | mg/L |
| 48 | Prealbumin | Prealbumin level for nutritional status | Others | continuous | mg/L |
| 49 | Albumin | Serum albumin levels | Liver | continuous | g/L |
| 50 | AGR | Albumin to globulin ratio | Liver | continuous | (0 - 1) |
| 51 | AST | Aspartate transferase | Liver | continuous | U/L |
| 52 | ALT | Alanine aminotransferase | Liver | continuous | U/L |
| 53 | ALP | Alkaline phosphatase | Liver | continuous | U/L |
| 54 | GGT | Gamma-glutamyl transferase | Liver | continuous | U/L |
| 55 | GMCSF | Granulocyte-macrophage colony- stimulating factor | Cytokines | continuous | pg/mL |
| 56 | IFNGamma | Interferon-γ | Cytokines | continuous | pg/mL |
| 57 | IL1Beta | Interleukin-1β | Cytokines | continuous | pg/mL |
| 58 | IL1RA | Interleukin-1 receptor antagonist | Cytokines | continuous | pg/mL |
| 59 | IL2 | Interleukin-2 | Cytokines | continuous | pg/mL |
| 60 | IL4 | Interleukin-4 | Cytokines | continuous | pg/mL |
| 61 | IL5 | Interleukin-5 | Cytokines | continuous | pg/mL |
| 62 | IL6 | Interleukin-6 | Cytokines | continuous | pg/mL |
| 63 | IL8 | Interleukin-8 | Cytokines | continuous | pg/mL |
| 64 | IL10 | Interleukin-10 | Cytokines | continuous | pg/mL |
| 65 | IL12p40 | Interleukin-12 p40 | Cytokines | continuous | pg/mL |
| 66 | IL12p70 | Interleukin-12 p70 | Cytokines | continuous | pg/mL |
| 67 | IL13 | Interleukin-13 | Cytokines | continuous | pg/mL |
| 68 | MCP1 | Monocyte chemoattractant protein-1 | Cytokines | continuous | pg/mL |
| 69 | TNFAlpha | Tumour necrosis factor-α | Cytokines | continuous | pg/mL |

# Section I: RBAC and Tryptophan

## Research question

In this section, we are trying to answer the following research question:


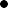
 *In cancer patients under active treatment, could RBAC supplementation affect the levels of tryptophan, kynurenine, and kynurenine-to-tryptophan ratio (KTR)?*

## RM ANOVA

Repeated measure ANOVA is used to compare between-group differences over multiple time points. In this study, the dependent variables (DV) of interest are Trp, Kyn, and KTR. We are interested in the effects of these variables: Group, Week and the interaction of Group * Week. Box-Cox transformation is applied to the DVs before running the RM-ANOVA test to ensure normality.

*# Loop from columns 5 to 7 of the data frame to run Two-way repeated measures ANOVA*

i_offset = 5

i_end = 7

cols <- c(i_offset:i_end) mdata <- MyData

res.aov <- list()

**for** (i **in** cols) {

x <- colnames(mdata[,i]) colnames(mdata)[i] = c("DV") mdata_t <- subset(mdata, !is.na(DV))

*# use boxcox transformation to ensure normality*

mdata_t$DV <- BoxCox(mdata_t$DV, "auto")

res.aov[[i-i_offset+1]] <- mdata_t %>% anova_test(DV ~ Group*Week+ Error(ID/(Week)) ) colnames(mdata)[i] = x

res.aov[[i-i_offset+1]][length(res.aov[[i-i_offset+1]]) + 1] = paste(x, "~ Group * Week + Error(ID/(Week))")

names(res.aov[[i-i_offset+1]])[length(res.aov[[i-i_offset+1]])] = "Formula" names(res.aov)[[i-i_offset+1]] = x

}

res.aov

## $Trp

## ANOVA Table (type III tests) ##

## $ANOVA

| ## |  | Effect | DFn | DFd | F | p | p<.05 | ges |
| --- | --- | --- | --- | --- | --- | --- | --- | --- |
| ## | 1 | Group | 1 | 12 | 0.106 | 0.750 |  | 0.007 |
| ## | 2 | Week | 4 | 48 | 0.105 | 0.980 |  | 0.002 |
| ## | 3 | Group:Week | 4 | 48 | 1.610 | 0.187 |  | 0.033 |
| ## |  |  |  |  |  |  |  |  |

## $`Mauchly's Test for Sphericity` ## Effect W p p<.05 ## 1 Week 0.328 0.241

## 2 Group:Week 0.328 0.241 ##

## $`Sphericity Corrections`

| ## | Effect | GGe | DF[GG] | p[GG] | p[GG]<.05 | HFe | DF[HF] | p[HF] |
| --- | --- | --- | --- | --- | --- | --- | --- | --- |
| ## 1 | Week | 0.667 | 2.67, 32.04 | 0.943 |  | 0.876 | 3.51, 42.06 | 0.971 |
| ## 2 | Group:Week | 0.667 | 2.67, 32.04 | 0.210 |  | 0.876 | 3.51, 42.06 | 0.195 |
| ## | p[HF]<.05 |  |  |  |  |  |  |  |

## 1

## 2 ##

## $Formula

## [1] "Trp ~ Group * Week + Error(ID/(Week))" ##

##

## $Kyn

## ANOVA Table (type III tests) ##

## $ANOVA

| ## |  | Effect | DFn | DFd | F | p | p<.05 | ges |
| --- | --- | --- | --- | --- | --- | --- | --- | --- |
| ## | 1 | Group | 1 | 12 | 0.123 | 0.732 |  | 0.007 |
| ## | 2 | Week | 4 | 48 | 2.154 | 0.088 |  | 0.049 |
| ## | 3 | Group:Week | 4 | 48 | 0.881 | 0.483 |  | 0.021 |
| ## |  |  |  |  |  |  |  |  |

| ## | $`Mauchly's Test for Sphericity` |  | | |
| --- | --- | --- | --- | --- |
| ##  ## | Effect W p p<.05  1 Week 0.253 0.116 |  |  |  |
| ##  ## | 2 Group:Week 0.253 0.116 |  |  |  |
| ## | $`Sphericity Corrections` |  |  |  |
| ## | Effect GGe DF[GG] p[GG] p[GG]<.05 | HFe | DF[HF] | p[HF] |
| ## | 1 Week 0.731 2.92, 35.07 0.112 | 0.991 | 3.96, 47.57 | 0.089 |
| ##  ## | 2 Group:Week 0.731 2.92, 35.07 0.458  p[HF]<.05 | 0.991 | 3.96, 47.57 | 0.482 |
| ## | 1 |  | | |
| ## | 2 |  |  |  |
| ## |  |  |  |  |
| ##  ## | $Formula  [1] "Kyn ~ Group * Week + Error(ID/(Week))" |  |  |  |
| ##  ## |  |  |  |  |
| ##  ## | $KTR  ANOVA Table (type III tests) |  |  |  |
| ## |  |  |  |  |

| ## |  | Effect | DFn | DFd | F | p | p<.05 | ges |
| --- | --- | --- | --- | --- | --- | --- | --- | --- |
| ## | 1 | Group | 1 | 12 | 0.237 | 0.635 |  | 0.013 |
| ## | 2 | Week | 4 | 48 | 2.864 | 0.033 | * | 0.074 |
| ## | 3 | Group:Week | 4 | 48 | 0.844 | 0.505 |  | 0.023 |
| ## |  |  |  |  |  |  |  |  |

| ## | $`Mauchly's Test for Sphericity` |  | | |
| --- | --- | --- | --- | --- |
| ## | Effect W p p<.05 |  |  |  |
| ##  ## | 1 Week 0.148 0.02 *  2 Group:Week 0.148 0.02 * |  |  |  |
| ##  ## | $`Sphericity Corrections` |  |  |  |
| ## | Effect GGe DF[GG] p[GG] p[GG]<.05 | HFe | DF[HF] | p[HF] |
| ## | 1 Week 0.584 2.34, 28.02 0.066 | 0.734 | 2.93, 35.21 | 0.052 |
| ##  ## | 2 Group:Week 0.584 2.34, 28.02 0.457  p[HF]<.05 | 0.734 | 2.93, 35.21 | 0.477 |
| ## | 1 |  |  |  |
| ##  ## | 2 |  |  |  |
| ##  ## | $Formula  [1] "KTR ~ Group * Week + Error(ID/(Week))" |  |  |  |

#### Note:

## $ANOVA

No significant difference is detected for Trp and Kyn measures. However, the results of the RM-ANOVA of KTR show a marginally significant difference over time (F[4,48] = 2.864, *p* = 0.052, eta2[g] = 0.074). The *p*-value reported is p[HF] after sphericity corrections

## Pairwise comparisons

Although no significant differences are detected, we continue performing pairwise comparisons between groups over time to check for significance.

*# Make sure we include only the DVs of interest*

sig.dv = c("Trp", "Kyn", "KTR") sig.cols <- c()

i = 1

**for** (w **in** sig.dv) {

sig.cols = c(sig.cols, which(colnames(mdata) == w) - i_offset + 1) i <- i+1

}

### Between treatment groups

*# Pairwise comparisons between treatment groups*

pwc <- list() i = 1

**for** (g **in** sig.cols) {

index <- i_offset + g -1

x <- colnames(mdata[,index]) colnames(mdata)[index] = c("DV") pwc[[i]] <- mdata %>%

group_by(Week) %>% pairwise_t_test(

DV ~ Group, paired = FALSE,

p.adjust.method = "fdr")

pwc[[i]]$.y. = x

names(pwc)[[i]] = x colnames(mdata)[index] = x i <- i+1

}

kable(pwc[[1]])

| **Week** | **.y.** | **group1** | **group2** | **n1** | **n2** | **p** | **p.signif** | **p.adj** | **p.adj.signif** |
| --- | --- | --- | --- | --- | --- | --- | --- | --- | --- |
| 0 | Trp | Placebo | RBAC | 10 | 9 | 0.567 | ns | 0.567 | ns |
| 6 | Trp | Placebo | RBAC | 8 | 7 | 0.446 | ns | 0.446 | ns |
| 12 | Trp | Placebo | RBAC | 9 | 7 | 0.812 | ns | 0.812 | ns |
| 18 | Trp | Placebo | RBAC | 8 | 7 | 0.275 | ns | 0.275 | ns |
| 24 | Trp | Placebo | RBAC | 8 | 7 | 0.686 | ns | 0.686 | ns |

kable(pwc[[2]])

| **Week** | **.y.** | **group1** | **group2** | **n1** | **n2** | **p** | **p.signif** | **p.adj** | **p.adj.signif** |
| --- | --- | --- | --- | --- | --- | --- | --- | --- | --- |
| 0 | Kyn | Placebo | RBAC | 10 | 9 | 0.483 | ns | 0.483 | ns |
| 6 | Kyn | Placebo | RBAC | 8 | 7 | 0.113 | ns | 0.113 | ns |
| 12 | Kyn | Placebo | RBAC | 9 | 7 | 0.307 | ns | 0.307 | ns |
| 18 | Kyn | Placebo | RBAC | 8 | 7 | 0.890 | ns | 0.890 | ns |
| 24 | Kyn | Placebo | RBAC | 8 | 7 | 0.492 | ns | 0.492 | ns |

kable(pwc[[3]])

| **Week** | **.y.** | **group1** | **group2** | **n1** | **n2** | **p** | **p.signif** | **p.adj** | **p.adj.signif** |
| --- | --- | --- | --- | --- | --- | --- | --- | --- | --- |
| 0 | KTR | Placebo | RBAC | 10 | 9 | 0.839 | ns | 0.839 | ns |
| 6 | KTR | Placebo | RBAC | 8 | 7 | 0.634 | ns | 0.634 | ns |
| 12 | KTR | Placebo | RBAC | 9 | 7 | 0.310 | ns | 0.310 | ns |
| 18 | KTR | Placebo | RBAC | 8 | 7 | 0.258 | ns | 0.258 | ns |

**Week .y. group1 group2 n1 n2 p p.signif p.adj p.adj.signif**

| 24 | KTR | Placebo | RBAC | 8 | 7 | 0.666 | ns | 0.666 | ns |
| --- | --- | --- | --- | --- | --- | --- | --- | --- | --- |

### Between time points

*# Pairwise comparisons between timepoints*

pwc1 <- list() i = 1

**for** (g **in** sig.cols) {

index <- i_offset + g -1

x <- colnames(mdata[,index]) colnames(mdata)[index] = c("DV") pwc1[[i]] <- mdata %>%

pairwise_t_test(

DV ~ Week, paired = FALSE, p.adjust.method = "fdr")

pwc1[[i]]$.y. = x

names(pwc1)[[i]] = x colnames(mdata)[index] = x i <- i+1

}

kable(pwc1[[1]])

| **.y.** | **group1** | **group2** | **n1** | **n2** | **p** | **p.signif** | **p.adj** | **p.adj.signif** |
| --- | --- | --- | --- | --- | --- | --- | --- | --- |
| Trp | 0 | 6 | 19 | 15 | 0.943 | ns | 0.951 | ns |
| Trp | 0 | 12 | 19 | 16 | 0.741 | ns | 0.951 | ns |
| Trp | 6 | 12 | 15 | 16 | 0.703 | ns | 0.951 | ns |
| Trp | 0 | 18 | 19 | 15 | 0.941 | ns | 0.951 | ns |
| Trp | 6 | 18 | 15 | 15 | 0.890 | ns | 0.951 | ns |
| Trp | 12 | 18 | 16 | 15 | 0.809 | ns | 0.951 | ns |
| Trp | 0 | 24 | 19 | 15 | 0.697 | ns | 0.951 | ns |
| Trp | 6 | 24 | 15 | 15 | 0.663 | ns | 0.951 | ns |
| Trp | 12 | 24 | 16 | 15 | 0.951 | ns | 0.951 | ns |
| Trp | 18 | 24 | 15 | 15 | 0.766 | ns | 0.951 | ns |

kable(pwc1[[2]])

| **.y.** | **group1** | **group2** | **n1** | **n2** | **p** | **p.signif** | **p.adj** | **p.adj.signif** |
| --- | --- | --- | --- | --- | --- | --- | --- | --- |
| Kyn | 0 | 6 | 19 | 15 | 0.787 | ns | 0.972 | ns |
| Kyn | 0 | 12 | 19 | 16 | 0.301 | ns | 0.787 | ns |
| Kyn | 6 | 12 | 15 | 16 | 0.472 | ns | 0.787 | ns |
| Kyn | 0 | 18 | 19 | 15 | 0.279 | ns | 0.787 | ns |
| Kyn | 6 | 18 | 15 | 15 | 0.440 | ns | 0.787 | ns |

**.y. group1 group2 n1 n2 p p.signif p.adj p.adj.signif**

| Kyn | 12 | 18 | 16 | 15 | 0.948 | ns | 0.972 | ns |
| --- | --- | --- | --- | --- | --- | --- | --- | --- |
| Kyn | 0 | 24 | 19 | 15 | 0.263 | ns | 0.787 | ns |
| Kyn | 6 | 24 | 15 | 15 | 0.420 | ns | 0.787 | ns |
| Kyn | 12 | 24 | 16 | 15 | 0.919 | ns | 0.972 | ns |
| Kyn | 18 | 24 | 15 | 15 | 0.972 | ns | 0.972 | ns |

kable(pwc1[[3]])

| **.y.** | **group1** | **group2** | **n1** | **n2** | **p** | **p.signif** | **p.adj** | **p.adj.signif** |
| --- | --- | --- | --- | --- | --- | --- | --- | --- |
| KTR | 0 | 6 | 19 | 15 | 0.848 | ns | 0.942 | ns |
| KTR | 0 | 12 | 19 | 16 | 0.527 | ns | 0.942 | ns |
| KTR | 6 | 12 | 15 | 16 | 0.680 | ns | 0.942 | ns |
| KTR | 0 | 18 | 19 | 15 | 0.369 | ns | 0.942 | ns |
| KTR | 6 | 18 | 15 | 15 | 0.503 | ns | 0.942 | ns |
| KTR | 12 | 18 | 16 | 15 | 0.789 | ns | 0.942 | ns |
| KTR | 0 | 24 | 19 | 15 | 0.517 | ns | 0.942 | ns |
| KTR | 6 | 24 | 15 | 15 | 0.666 | ns | 0.942 | ns |
| KTR | 12 | 24 | 16 | 15 | 0.979 | ns | 0.979 | ns |
| KTR | 18 | 24 | 15 | 15 | 0.812 | ns | 0.942 | ns |

#### Note:

Again, we confirm no significant difference has been detected for Trp, Kyn, and KTR in pairwise comparisons between groups over time.

## Data summary and visualisation

### Summary statistics for Trp, Kyn, and KTR (between groups and over time)

Stat_Summary <- list() i = 1

**for** (g **in** sig.cols) { index <- i_offset + g -1

x <- colnames(mdata[,index]) colnames(mdata)[index] = c("DV")

Stat_Summary[[i]] <- mdata %>% group_by(Group, Week) %>%

get_summary_stats(DV, type = "common", show=c("mean", "sd", "se", "c

i")) %>%

left_join(pwc[[i]][,c(1,9,10)], by = join_by(Week == Week))

Stat_Summary[[i]]$variable = x colnames(mdata)[index] = x names(Stat_Summary)[[i]] = x

i <- i+1

}

kable(Stat_Summary[[1]])

| **Group** | **Week** | **variable** | **n** | **mean** | **sd** | **se** | **ci** | **p.adj** | **p.adj.signif** |
| --- | --- | --- | --- | --- | --- | --- | --- | --- | --- |
| Placebo | 0 | Trp | 10 | 53.486 | 11.199 | 3.541 | 8.011 | 0.567 | ns |
| Placebo | 6 | Trp | 8 | 48.717 | 14.946 | 5.284 | 12.495 | 0.446 | ns |
| Placebo | 12 | Trp | 9 | 53.994 | 14.062 | 4.687 | 10.809 | 0.812 | ns |
| Placebo | 18 | Trp | 8 | 56.363 | 17.878 | 6.321 | 14.946 | 0.275 | ns |
| Placebo | 24 | Trp | 8 | 54.965 | 16.277 | 5.755 | 13.608 | 0.686 | ns |
| RBAC | 0 | Trp | 9 | 49.800 | 16.142 | 5.381 | 12.408 | 0.567 | ns |
| RBAC | 6 | Trp | 7 | 54.456 | 13.067 | 4.939 | 12.085 | 0.446 | ns |
| RBAC | 12 | Trp | 7 | 52.396 | 11.687 | 4.417 | 10.808 | 0.812 | ns |
| RBAC | 18 | Trp | 7 | 47.214 | 12.159 | 4.596 | 11.245 | 0.275 | ns |
| RBAC | 24 | Trp | 7 | 52.043 | 9.708 | 3.669 | 8.978 | 0.686 | ns |

kable(Stat_Summary[[2]])

| **Group** | **Week** | **variable** | **n** | **mean** | **sd** | **se** | **ci** | **p.adj** | **p.adj.signif** |
| --- | --- | --- | --- | --- | --- | --- | --- | --- | --- |
| Placebo | 0 | Kyn | 10 | 3.654 | 1.227 | 0.388 | 0.878 | 0.483 | ns |
| Placebo | 6 | Kyn | 8 | 3.257 | 0.832 | 0.294 | 0.696 | 0.113 | ns |
| Placebo | 12 | Kyn | 9 | 3.654 | 1.048 | 0.349 | 0.805 | 0.307 | ns |
| Placebo | 18 | Kyn | 8 | 3.872 | 1.143 | 0.404 | 0.955 | 0.890 | ns |
| Placebo | 24 | Kyn | 8 | 4.199 | 2.075 | 0.734 | 1.735 | 0.492 | ns |
| RBAC | 0 | Kyn | 9 | 3.277 | 1.044 | 0.348 | 0.802 | 0.483 | ns |
| RBAC | 6 | Kyn | 7 | 3.959 | 0.756 | 0.286 | 0.699 | 0.113 | ns |
| RBAC | 12 | Kyn | 7 | 4.185 | 0.913 | 0.345 | 0.845 | 0.307 | ns |

**Group Week variable n mean sd se ci p.adj p.adj.signif**

| RBAC | 18 | Kyn | 7 | 3.962 | 1.319 | 0.498 | 1.220 | 0.890 | ns |
| --- | --- | --- | --- | --- | --- | --- | --- | --- | --- |
| RBAC | 24 | Kyn | 7 | 3.620 | 0.623 | 0.235 | 0.576 | 0.492 | ns |

kable(Stat_Summary[[3]])

| **Group** | **Week** | **variable** | **n** | **mean** | **sd** | **se** | **ci** | **p.adj** | **p.adj.signif** |
| --- | --- | --- | --- | --- | --- | --- | --- | --- | --- |
| Placebo | 0 | KTR | 10 | 0.072 | 0.034 | 0.011 | 0.024 | 0.839 | ns |
| Placebo | 6 | KTR | 8 | 0.070 | 0.020 | 0.007 | 0.016 | 0.634 | ns |
| Placebo | 12 | KTR | 9 | 0.071 | 0.022 | 0.007 | 0.017 | 0.310 | ns |
| Placebo | 18 | KTR | 8 | 0.072 | 0.020 | 0.007 | 0.016 | 0.258 | ns |
| Placebo | 24 | KTR | 8 | 0.080 | 0.040 | 0.014 | 0.033 | 0.666 | ns |
| RBAC | 0 | KTR | 9 | 0.069 | 0.023 | 0.008 | 0.018 | 0.839 | ns |
| RBAC | 6 | KTR | 7 | 0.074 | 0.014 | 0.005 | 0.013 | 0.634 | ns |
| RBAC | 12 | KTR | 7 | 0.083 | 0.023 | 0.009 | 0.021 | 0.310 | ns |
| RBAC | 18 | KTR | 7 | 0.086 | 0.028 | 0.010 | 0.025 | 0.258 | ns |
| RBAC | 24 | KTR | 7 | 0.072 | 0.020 | 0.008 | 0.018 | 0.666 | ns |

Data plots for Trp, Kyn, and KTR (between groups and over time)

pd <- position_dodge(0.1) *# move them .05 to the left and right*

*# First, generate the individual plot*

Stat_Plot <- list()

**for** (n **in** c(1:length(Stat_Summary))) {

Stat_Plot[[n]] <-ggplot(Stat_Summary[[n]], aes(x=Week, y=mean, colour=Group)) + ylab(sig.dv[[n]]) +

geom_errorbar(aes(ymin=mean-se, ymax=mean+se), width=.5, position=pd) +

geom_line(position=pd) + geom_point(position=pd) + scale_x_continuous(breaks=seq(0,30,6))

names(Stat_Plot)[[n]] = names(Stat_Summary)[[n]]

}

*# Then, combined the plots into one*

figure <- ggarrange(Stat_Plot[[1]], Stat_Plot[[2]], Stat_Plot[[3]], labels = c("(I)", "(II)", "(III)"),

ncol = 1, nrow = 3, common.legend = TRUE)

figure


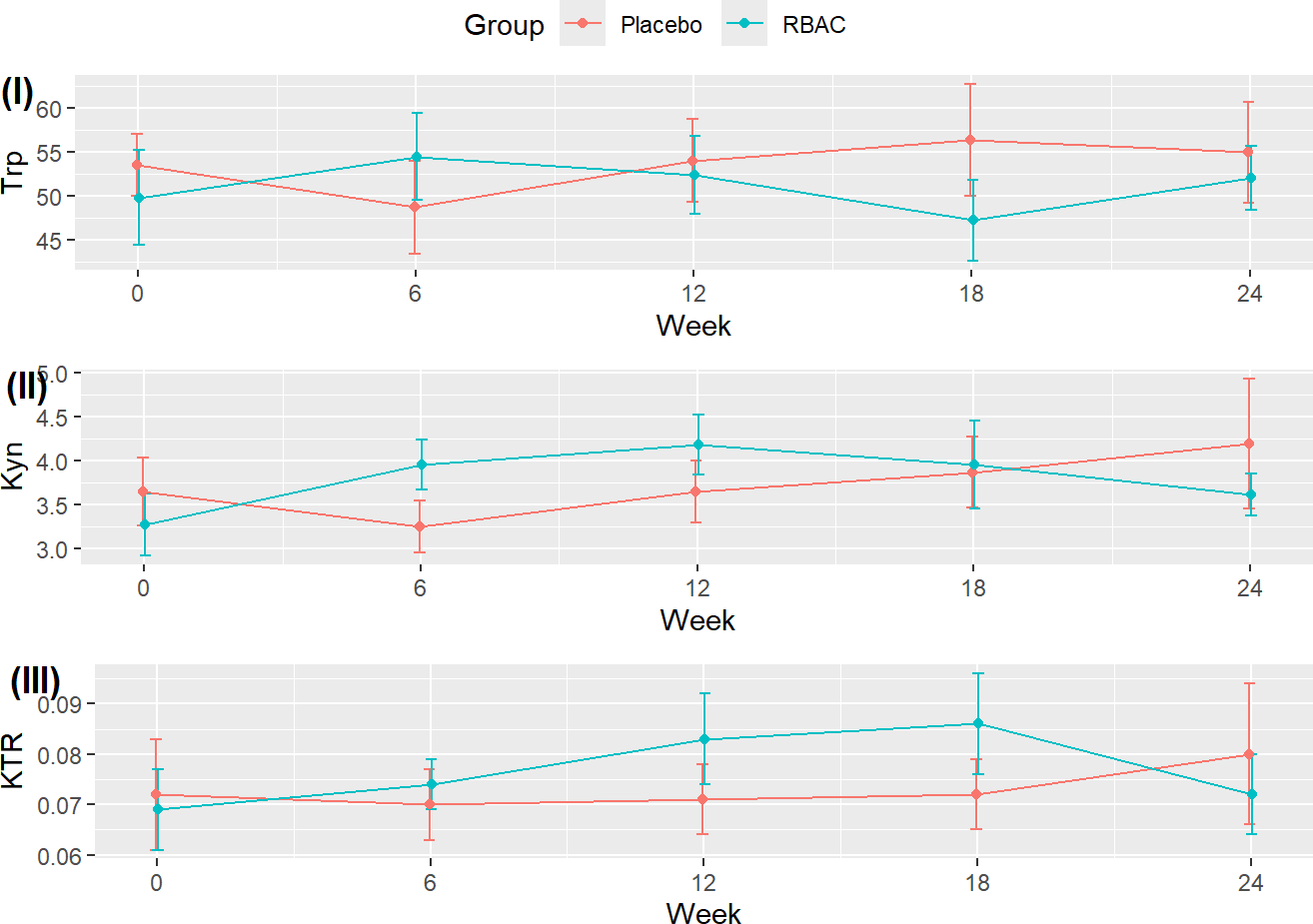


## Adjusted analysis with ANCOVA

Age at baseline has been identified as a covariate since significant between-group differences exist. We will analyse whether RBAC affects Trp, Kyn, and KTR, adjusting for age.

*# Loop from column 6 to the last column of the data frame to run repeated measures ANOVA with Age as a covariate*

res.ancov <- list()

**for** (i **in** cols) {

x <- colnames(mdata[,i]) colnames(mdata)[i] = c("DV") mdata_t <- subset(mdata, !is.na(DV))

*# use boxcox transformation to ensure normality*

mdata_t$DV <- BoxCox(mdata_t$DV, "auto")

res.ancov[[i-i_offset+1]] <- mdata_t %>% anova_test(DV ~ Group*Week+ Error(ID/(Week)), co variate = c(AgeAtBaseline), type=3)

colnames(mdata)[i] = x

res.ancov[[i-i_offset+1]][length(res.ancov[[i-i_offset+1]]) + 1] = paste(x, "~ Group * We ek + Error(ID/(Week))", ", covariate = AgeAtBaseline")

names(res.ancov[[i-i_offset+1]])[length(res.ancov[[i-i_offset+1]])] = "Formula" names(res.ancov)[[i-i_offset+1]] = x

}

res.ancov

## $Trp

## ANOVA Table (type III tests) ##

## $ANOVA

| ## |  | Effect | DFn | DFd | F | p | p<.05 | ges |
| --- | --- | --- | --- | --- | --- | --- | --- | --- |
| ## | 1 | AgeAtBaseline | 1 | 11 | 0.757 | 0.403 |  | 0.050 |
| ## | 2 | Group | 1 | 11 | 0.119 | 0.737 |  | 0.008 |
| ## | 3 | Week | 4 | 44 | 1.490 | 0.221 |  | 0.032 |
| ## | 4 | AgeAtBaseline:Week | 4 | 44 | 1.453 | 0.233 |  | 0.031 |
| ##  ## | 5 | Group:Week | 4 | 44 | 1.136 | 0.352 |  | 0.024 |
| ## $`Mauchly's Test for Sphericity` | | | | | | | | |

| ## | Effect W | p p<.05 |  | | | | |
| --- | --- | --- | --- | --- | --- | --- | --- |
| ## | 1 Week 0.208 | 0.102 |  |  |  |  |  |
| ## | 2 AgeAtBaseline:Week 0.208 | 0.102 |  |  |  |  |  |
| ## | 3 Group:Week 0.208 | 0.102 |  |  |  |  |  |
| ##  ## | $`Sphericity Corrections` |  |  |  |  |  |  |
| ## | Effect GGe | DF[GG] | p[GG] | p[GG]<.05 | HFe | DF[HF] | p[HF] |
| ## | 1 Week 0.575 | 2.3, 25.31 | 0.244 |  | 0.736 2.94, 32.38 | | 0.236 |
| ## | 2 AgeAtBaseline:Week 0.575 | 2.3, 25.31 | 0.253 |  | 0.736 2.94, 32.38 | | 0.246 |
| ## | 3 Group:Week 0.575 | 2.3, 25.31 | 0.343 |  | 0.736 2.94, 32.38 | | 0.349 |
| ## | p[HF]<.05 |  |  |  |  |  |  |

## 1

## 2

## 3 ##

## $Formula

## [1] "Trp ~ Group * Week + Error(ID/(Week)) , covariate = AgeAtBaseline" ##

##

## $Kyn

## ANOVA Table (type III tests) ##

## $ANOVA

| ## |  | Effect | DFn | DFd | F | p | p<.05 | ges |
| --- | --- | --- | --- | --- | --- | --- | --- | --- |
| ## | 1 | AgeAtBaseline | 1 | 11 | 0.360 | 0.561 |  | 0.023 |
| ## | 2 | Group | 1 | 11 | 0.429 | 0.526 |  | 0.027 |
| ## | 3 | Week | 4 | 44 | 0.866 | 0.492 |  | 0.021 |
| ## | 4 | AgeAtBaseline:Week | 4 | 44 | 1.019 | 0.408 |  | 0.025 |
| ## | 5 | Group:Week | 4 | 44 | 0.837 | 0.509 |  | 0.021 |
| ## |  |  |  |  |  |  |  |  |
| ## $`Mauchly's Test for Sphericity` | | | | | | | | |

| ##  ## | Effect W  1 Week 0.214 | p p<.05  0.11 |  | | | | |
| --- | --- | --- | --- | --- | --- | --- | --- |
| ## | 2 AgeAtBaseline:Week 0.214 | 0.11 |  |  |  |  |  |
| ## | 3 Group:Week 0.214 | 0.11 |  |  |  |  |  |
| ## |  |  |  |  |  |  |  |
| ##  ## | $`Sphericity Corrections`  Effect GGe | DF[GG] | p[GG] | p[GG]<.05 | HFe | DF[HF] | p[HF] |
| ## | 1 Week 0.692 | 2.77, 30.43 | 0.462 |  | 0.947 3.79, 41.67 | | 0.488 |
| ## | 2 AgeAtBaseline:Week 0.692 | 2.77, 30.43 | 0.393 |  | 0.947 3.79, 41.67 | | 0.406 |
| ##  ## | 3 Group:Week 0.692  p[HF]<.05 | 2.77, 30.43 | 0.476 |  | 0.947 3.79, 41.67 | | 0.504 |
| ## | 1 |  |  |  |  |  |  |

## 2

## 3 ##

## $Formula

## [1] "Kyn ~ Group * Week + Error(ID/(Week)) , covariate = AgeAtBaseline" ##

##

## $KTR

## ANOVA Table (type III tests) ##

## $ANOVA

| ## |  | Effect | DFn | DFd | F | p | p<.05 | ges |
| --- | --- | --- | --- | --- | --- | --- | --- | --- |
| ## | 1 | AgeAtBaseline | 1 | 11 | 0.100 | 0.758 |  | 0.006 |
| ## | 2 | Group | 1 | 11 | 0.018 | 0.895 |  | 0.001 |
| ## | 3 | Week | 4 | 44 | 1.173 | 0.336 |  | 0.033 |
| ## | 4 | AgeAtBaseline:Week | 4 | 44 | 1.140 | 0.350 |  | 0.032 |
| ## | 5 | Group:Week | 4 | 44 | 1.654 | 0.178 |  | 0.045 |
| ## |  |  |  |  |  |  |  |  |
| ## $`Mauchly's Test for Sphericity` | | | | | | | | |

| ##  ## | Effect W  1 Week 0.137 | p  0.03 | p<.05  * |  | | | | |
| --- | --- | --- | --- | --- | --- | --- | --- | --- |
| ## | 2 AgeAtBaseline:Week 0.137 | 0.03 | * |  |  |  |  |  |
| ## | 3 Group:Week 0.137 | 0.03 | * |  |  |  |  |  |
| ## |  |  |  |  |  |  |  |  |
| ##  ## | $`Sphericity Corrections`  Effect GGe |  | DF[GG] | p[GG] | p[GG]<.05 | HFe | DF[HF] | p[HF] |
| ## | 1 Week 0.568 | 2.27, 24.97 | | 0.331 |  | 0.723 2.89, 31.81 | | 0.335 |
| ## | 2 AgeAtBaseline:Week 0.568 | 2.27, 24.97 | | 0.341 |  | 0.723 2.89, 31.81 | | 0.347 |
| ##  ## | 3 Group:Week 0.568  p[HF]<.05 | 2.27, 24.97 | | 0.209 |  | 0.723 2.89, 31.81 | | 0.198 |

## 1

## 2

## 3 ##

## $Formula

## [1] "KTR ~ Group * Week + Error(ID/(Week)) , covariate = AgeAtBaseline"

#### Note:

Adjusted analysis with age as a covariate also shows no significant difference detected for Trp, Kyn, and KTR in pairwise comparisons between groups over time.

## Conclusion

The null hypothesis that RBAC supplementation has **NO effect** on the levels of tryptophan, kynurenine, and KTR over time cannot be rejected. The ANCOVA analysis also shows **no significant differences** between groups for all three parameters.

# Section II: Tryptophan and QoL outcomes

## Research question

In this section, we are trying to answer the following research question:


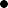
 *Are tryptophan, kynurenine, and KTR related to the reported QoL outcomes of the patients?*

## Spearman’s rank correlations

*# Perform the correlation tests*

TQ_x <- MyData %>% dplyr::select(Trp, Kyn, KTR ) TQ_y <- MyData[,8:23] *# Only the QLQ-C30 outcomes*

TQ_z <- corr.test(TQ_x,TQ_y, method="spearman", adjust="fdr")

*# Create star strings based on the adjusted p values*

TQ_m <- cbind(t(TQ_z$r), t(TQ_z$p.adj))

colnames(TQ_m) <- c("Trp", "Kyn", "KTR", "p.adj.Trp", "p.adj.Kyn", "p.adj.KTR") TQ_df <-as.data.frame(TQ_m)

TQ_df <- TQ_df %>% mutate (Trp.star = case_when(

p.adj.Trp <= 0.001 ~ paste(as.character(round(Trp,3)), "***") , p.adj.Trp <= 0.01 ~ paste(as.character(round(Trp,3)), "**"), p.adj.Trp <= 0.05 ~ paste(as.character(round(Trp,3)), "*"), TRUE ~ as.character(round(Trp,3))

))

TQ_df <- TQ_df %>% mutate (Kyn.star = case_when(

p.adj.Kyn <= 0.001 ~ paste(as.character(round(Kyn,3)), "***") , p.adj.Kyn <= 0.01 ~ paste(as.character(round(Kyn,3)), "**"), p.adj.Kyn <= 0.05 ~ paste(as.character(round(Kyn,3)), "*"), TRUE ~ as.character(round(Kyn,3))

))

TQ_df <- TQ_df %>% mutate (KTR.star = case_when(

p.adj.KTR <= 0.001 ~ paste(as.character(round(KTR,3)), "***") , p.adj.KTR <= 0.01 ~ paste(as.character(round(KTR,3)), "**"), p.adj.KTR <= 0.05 ~ paste(as.character(round(KTR,3)), "*"), TRUE ~ as.character(round(KTR,3))

))

TQ_df <- TQ_df %>% dplyr::select(Trp.star, Kyn.star, KTR.star ) TQ_star <- matrix(unlist(TQ_df), nrow=nrow(TQ_df),ncol=ncol(TQ_df)) colnames(TQ_star) <- c("Trp", "Kyn", "KTR")

rownames(TQ_star) <- rownames(TQ_df)

*# Plot a heatmap to display the correlation*

data <- t(TQ_z$r) my_cellnote <- TQ_star

colors = c(seq(-3,-2,length=100),seq(-2,0.5,length=100),seq(0.5,6,length=100)) my_palette <- colorRampPalette(c("yellow", "white", "green"))(n = 10) my_breaks <- seq(-1,1, 0.2)

heatmap.2( data, Rowv=NA, Colv=NA, col=my_palette, density.info="none", trace="none", cellnot e =my_cellnote, notecol="blue", key.title="Colour Key")


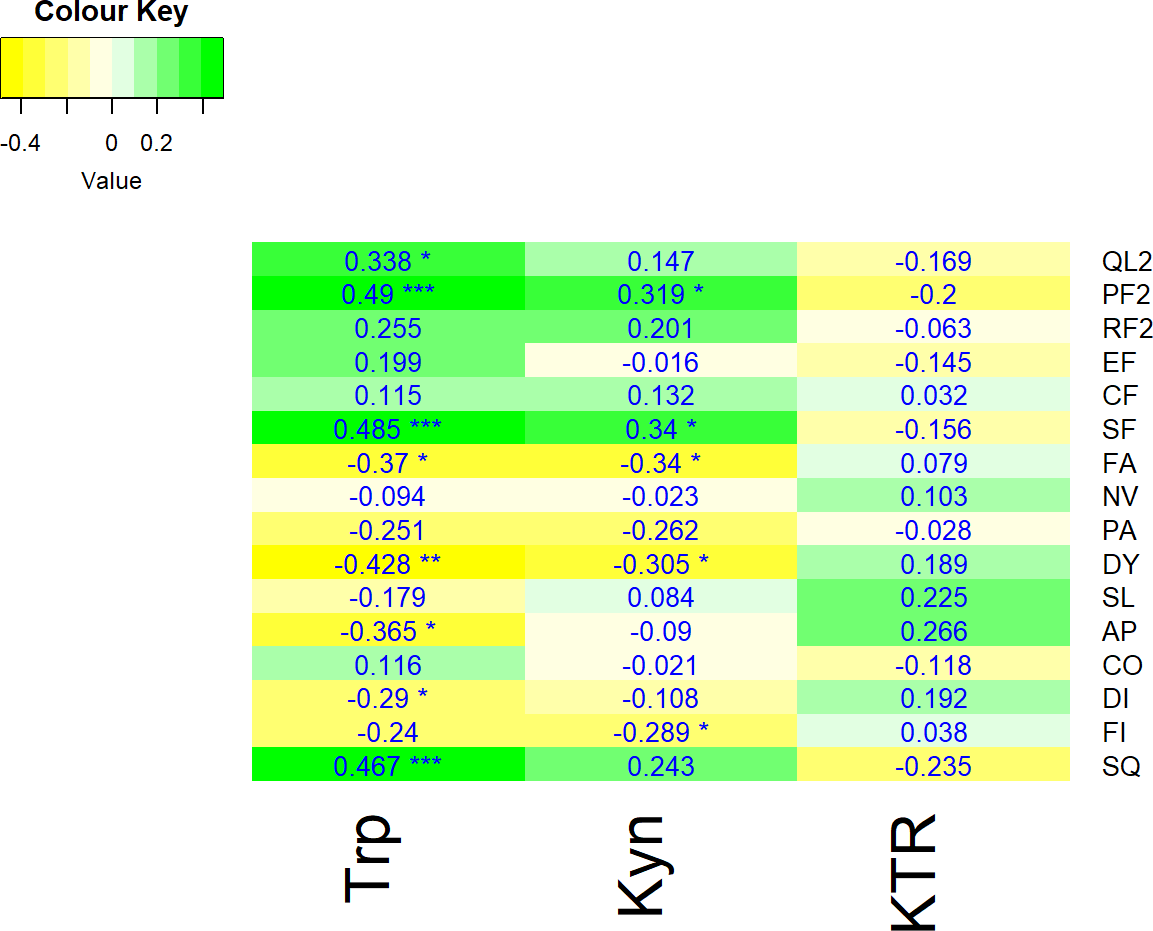


#### Significant findings:

**Trp** has significant **correlations**:

Positively with:


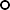
 global QoL (rs = 0.338, p ≤ 0.05)


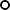
 physical functioning (rs = 0.49, p ≤ 0.001)
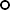
 social functioning (rs = 0.485, p ≤ 0.001)


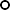
 overall QoL measured with SQ (rs = 0.467, p ≤ 0.001)

Negatively with:


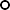
 fatigue (rs = -0.37, p ≤ 0.05)


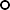
 dyspnoea (rs = -0.428, p ≤ 0.001)


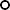
 appetite loss (rs = -0.365, p ≤ 0.05)
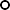
 diarrhoea (rs = -0.29, p ≤ 0.05)

**Kyn** also demonstrates the following significant **correlations**:

Positively with:


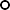
 physical functioning (rs = 0.319, p ≤ 0.05)
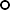
 social functioning (rs = 0.34, p ≤ 0.05)

Negatively with:


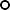
 fatigue (rs = -0.34, p ≤ 0.05)


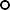
 dyspnoea (rs = -0.305, p ≤ 0.05)


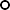
 financial impact (rs = -0.289, p ≤ 0.05)

**KTR** exhibited no significant correlations with any of the QoL outcome measures.

## Conclusion

Tryptophan and kynurenine levels, but not KTR, show correlations with some of the reported QoL outcomes of the patients. Specifically, only tryptophan shows a moderately strong correlation with overall QoL measured with SQ (rs = 0.467, p ≤ 0.001).

# Section III: Tryptophan vs. Other Significant Factors

## Research question

In this section, we are trying to answer the following research question:


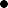
 *Are tryptophan, kynurenine, and KTR significant factors in predicting QoL compared to other blood markers for haematological, immune, inflammatory and nutritional markers?*

## Spearman’s rank correlations

To answer the research question, we must first identify other significant factors. We will perform Spearman’s rank correlations for all other blood markers and age.

*# Remove columns not to be analysed*

TQ_y <- cbind(TQ_x, MyData[,25:69]) *# Include only Trp, Kyn, KTR, age and all blood tests* TQ_x <- MyData %>% dplyr::select(SQ) *# We are interested only with SQ as the QoL outcome meas ure*

*# Peform Spearman correlation tests*

TQ_z <- corr.test(TQ_x,TQ_y, method = "spearman", adjust ="fdr")

*# Create star strings based on the adusted p values* TQ_m <- cbind(t(TQ_z$r), t(TQ_z$p.adj)) colnames(TQ_m) <- c("r.SQ", "p.adj.SQ")

TQ_df <-as.data.frame(TQ_m)

TQ_df <- TQ_df %>% mutate (SQ.star = case_when( p.adj.SQ <= 0.001 ~ "***" ,

p.adj.SQ <= 0.01 ~ "**",

p.adj.SQ <= 0.05 ~ "*", TRUE ~ ""

)) %>%

rownames_to_column()

*# Display the Spearman's correlation tests for all factors*

colnames(TQ_df) <- c("Factors", "r.SQ", "p.adj.SQ", "star")

TQ_df <- TQ_df %>% mutate(rSQ.star = paste(round(r.SQ,3), star )) %>% arrange(desc(r.SQ)) kable(TQ_df, caption = "Spearman's correlation tests for all factors.")

Spearman’s correlation tests for all factors.

| **Factors** | **r.SQ** | **p.adj.SQ** | **star** | **rSQ.star** |
| --- | --- | --- | --- | --- |
| Trp | 0.4670308 | 0.0007463 | *** | 0.467 *** |
| Haemoglobin | 0.4374224 | 0.0017434 | ** | 0.437 ** |
| Haematocrit | 0.4280228 | 0.0019016 | ** | 0.428 ** |

| RBC | 0.4104512 | 0.0025097 | ** | 0.41 ** |
| --- | --- | --- | --- | --- |
| Urea | 0.4101429 | 0.0025097 | ** | 0.41 ** |
| Chloride | 0.4010582 | 0.0028405 | ** | 0.401 ** |
| Prealbumin | 0.3928516 | 0.0031264 | ** | 0.393 ** |
| Albumin | 0.3671322 | 0.0067119 | ** | 0.367 ** |
| Lymphocytes | 0.3539538 | 0.0090403 | ** | 0.354 ** |
| TotalBilirubin | 0.3285957 | 0.0167443 | * | 0.329 * |
| Bicarbonate | 0.3088658 | 0.0251450 | * | 0.309 * |
| Creatinine | 0.3087962 | 0.0251450 | * | 0.309 * |
| MCP1 | 0.2694485 | 0.0662657 |  | 0.269 |
| AGR | 0.2598130 | 0.0746326 |  | 0.26 |
| IL4 | 0.2549073 | 0.0818666 |  | 0.255 |
| Kyn | 0.2425213 | 0.0977807 |  | 0.243 |
| ALT | 0.2321696 | 0.1099340 |  | 0.232 |
| Age | 0.2226388 | 0.1272591 |  | 0.223 |
| Sodium | 0.2126419 | 0.1479474 |  | 0.213 |
| GMCSF | 0.1952371 | 0.1925345 |  | 0.195 |
| IL10 | 0.1859407 | 0.2175885 |  | 0.186 |
| MCHC | 0.1754100 | 0.2360747 |  | 0.175 |
| IL12p40 | 0.1287861 | 0.4495409 |  | 0.129 |
| IL2 | 0.1080229 | 0.5310990 |  | 0.108 |
| IL13 | 0.1058490 | 0.5310990 |  | 0.106 |
| IL1RA | 0.1009109 | 0.5471852 |  | 0.101 |
| WBC | 0.0841950 | 0.5958887 |  | 0.084 |
| Basophils | 0.0497250 | 0.7735639 |  | 0.05 |
| Potassium | 0.0492872 | 0.7735639 |  | 0.049 |
| Eosinophils | 0.0469944 | 0.7735639 |  | 0.047 |
| MCH | 0.0066572 | 0.9557408 |  | 0.007 |
| TNFAlpha | 0.0066571 | 0.9557408 |  | 0.007 |
| IFNGamma | -0.0205201 | 0.9017255 |  | -0.021 |
| IL12p70 | -0.0218212 | 0.9017255 |  | -0.022 |
| Neutrophils | -0.0352247 | 0.8370973 |  | -0.035 |

| IL5 | -0.0665332 | 0.6944370 |  | -0.067 |
| --- | --- | --- | --- | --- |
| IL1Beta | -0.0837730 | 0.5958887 |  | -0.084 |
| IL8 | -0.0903690 | 0.5841594 |  | -0.09 |
| IL6 | -0.0968193 | 0.5579709 |  | -0.097 |
| AST | -0.1128625 | 0.5126381 |  | -0.113 |
| MCV | -0.1248857 | 0.4528451 |  | -0.125 |
| Monocytes | -0.1682842 | 0.2560304 |  | -0.168 |
| ALP | -0.1815783 | 0.2207745 |  | -0.182 |
| Platelet | -0.2041841 | 0.1662820 |  | -0.204 |
| KTR | -0.2348267 | 0.1092552 |  | -0.235 |
| RDW | -0.3519762 | 0.0090403 | ** | -0.352 ** |
| CRP | -0.3988671 | 0.0028405 | ** | -0.399 ** |
| GGT | -0.5361044 | 0.0000489 | *** | -0.536 *** |

*# Plot only the significant factors*

TQ_df.sig <- TQ_df %>% dplyr::filter(p.adj.SQ <= 0.05)

figure1 <- ggplot(TQ_df.sig, aes(x = reorder(Factors, r.SQ), y = r.SQ, fill=r.SQ)) + geom_bar(stat = "identity",

show.legend = FALSE) + xlab("Factors") +

ylab("Correlation Coefficient with SQ") + coord_flip() +

scale_fill_gradient2(

low = "red", mid = "white", high = "green", midpoint = 0

) + geom_text(aes(Factors,round(r.SQ,3), label=rSQ.star),hjust= 0.5, vjust=0.5, size=4, position = position_dodge(width = 1))

figure1


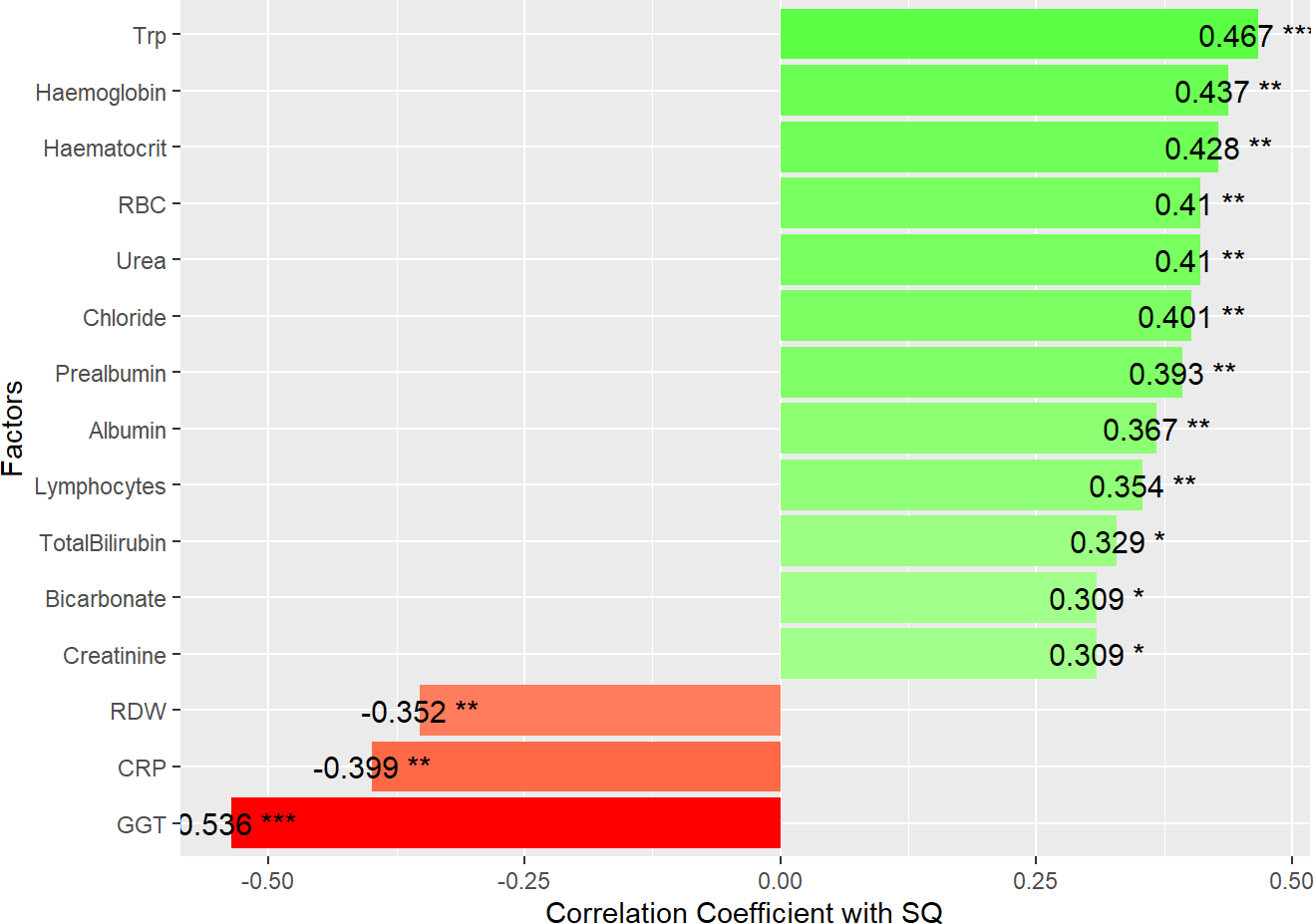


#### Notes :

Many blood markers positively affected SQ, including haematological (red blood cell count [RBC], haemoglobin, haematocrit), electrolytes (chloride, bilirubin, bicarbonate), liver function (urea), and immune and nutritional markers (lymphocyte, albumin, prealbumin). Among all, tryptophan is the most prominent positive factor. Factors negatively correlated with SQ are the inflammatory marker, CRP, red cell distribution width (RDW) and gamma-glutamyl transferase (GGT). None of the cytokines exhibit any significant correlations with SQ.

## Linear mixed model

### Full model

A linear mixed model catered for group-specific effects and a random slope for weeks is used to fit the data with SQ as the dependent outcome measure and all significant blood markers as fixed effects of the model.

*# Create a full model based on the significant factors only*

SQfull <- MyData %>% dplyr::select(ID, Week, Group, SQ, TQ_df.sig$Factors) %>% mutate(ID = a s.factor(ID))

*# Loop from to run BoxCox conversion to ensure normality*

n_offset = 4

cols <- c(n_offset:ncol(SQfull)) Lamda <- list()

**for** (i **in** cols) {

x <- colnames(SQfull[i]) colnames(SQfull)[i] = c("DV")

*# use boxcox transformation to ensure normality* lam <- round(BoxCox.lambda(SQfull$DV), 2) SQfull$DV <- BoxCox(SQfull$DV, lam)

Lamda[i-n_offset+1] <- lam colnames(SQfull)[i] = x names(Lamda)[i-n_offset+1] = x

}

*# Define the formula*

rand_eff <- c("Group")

rand_slope <- paste(TQ_df.sig$Factors,collapse=" + ") fixed_eff <- paste("Week", rand_slope, sep = " + ") fixed_eff <- rand_slope

formula <- paste("SQ ~", fixed_eff, "+ (1 + Week | ", rand_eff, ")")

print(formula)

## [1] "SQ ~ Trp + Haemoglobin + Haematocrit + RBC + Urea + Chloride + Prealbumin + Albumin + Lymphocytes + TotalBilirubin + Bicarbonate + Creatinine + RDW + CRP + GGT + (1 + Week | Gro up )"

*# Fit the linear mixed model*

SQ_full_model <- lmer(formula, REML = F, data = SQfull) sumSQ_full_model <- summary(SQ_full_model)

*# Display the results of the full model fit*

sumSQ_full_model

## Linear mixed model fit by maximum likelihood . t-tests use Satterthwaite's ## method [lmerModLmerTest]

## Formula: formula ## Data: SQfull ##

| ## | AIC | BIC | logLik | -2*log(L) | df.resid |
| --- | --- | --- | --- | --- | --- |
| ##  ## | 1194.3 | 1240.1 | -577.1 | 1154.3 | 53 |

## Scaled residuals:

## Min 1Q Median 3Q Max ## -2.86722 -0.62618 0.05503 0.71751 2.21248 ##

## Random effects:

| ##  ## | Groups Name Variance Std.Dev. Corr  Group (Intercept) 118446.0 344.16 |  |  |  |  |
| --- | --- | --- | --- | --- | --- |
| ## | Week 114.6 10.71 1.00 |  |  |  |  |
| ##  ## | Residual 396081.4 629.35  Number of obs: 73, groups: Group, 2 |  |  |  |  |
| ##  ## | Fixed effects: |  |  |  |  |
| ##  ## | Estimate Std. Error df  (Intercept) 1.499e+04 4.485e+04 7.221e+01 | t | value  0.334 | Pr(>\|t\|)  0.739105 |  |
| ## | Trp 7.428e+00 1.820e+00 7.160e+01 |  | 4.082 | 0.000115 | *** |
| ## | Haemoglobin 8.644e-02 1.614e-01 7.257e+01 |  | 0.536 | 0.593873 |  |
| ## | Haematocrit -3.345e+03 2.149e+04 7.094e+01 |  | -0.156 | 0.876743 |  |
| ## | RBC -2.763e+02 4.060e+03 7.180e+01 |  | -0.068 | 0.945929 |  |
| ## | Urea 2.074e+02 3.590e+02 7.044e+01 |  | 0.578 | 0.565282 |  |
| ## | Chloride 2.454e-01 3.586e-01 7.208e+01 |  | 0.684 | 0.496007 |  |
| ## | Prealbumin 1.570e+02 6.458e+02 6.927e+01 |  | 0.243 | 0.808662 |  |
| ## | Albumin 4.359e-01 6.470e-01 7.151e+01 |  | 0.674 | 0.502602 |  |
| ## | Lymphocytes -2.815e+02 1.077e+02 7.178e+01 |  | -2.614 | 0.010907 | * |
| ## | TotalBilirubin -8.552e+02 4.187e+02 7.294e+01 |  | -2.043 | 0.044690 | * |
| ## | Bicarbonate 2.263e+00 1.408e+00 7.046e+01 |  | 1.607 | 0.112553 |  |
| ## | Creatinine -2.063e+04 4.313e+04 6.895e+01 |  | -0.478 | 0.633891 |  |
| ## | RDW 1.603e+04 1.423e+04 7.172e+01 |  | 1.126 | 0.263731 |  |
| ## | CRP -1.360e+02 1.371e+02 7.066e+01 |  | -0.992 | 0.324550 |  |
| ##  ## | GGT -8.904e+03 3.122e+03 6.961e+01  --- |  | -2.852 | 0.005714 | ** |

## Signif. codes: 0 '***' 0.001 '**' 0.01 '*' 0.05 '.' 0.1 ' ' 1

## fit warnings:

## Some predictor variables are on very different scales: consider rescaling ## optimizer (nloptwrap) convergence code: 0 (OK)

## boundary (singular) fit: see help('isSingular')

*# Display the random effects of Group*

ranef(SQ_full_model)

## $Group

##

## Placebo ## RBAC

##

(Intercept)

Week

-438.6411 -13.646310

183.7605

5.716867

## with conditional variances for "Group"

#### Notes :

Among the factors, only tryptophan (*p* < 0.001), GGT (*p* = 0.006), lymphocyte (*p* = 0.011), and total bilirubin (*p*

= 0.045) are factors for predicting SQ with significant differences between groups.

### Stepwise reduction

We now perform automatic backward elimination of all effects of the full model. First, backward elimination of the random part is performed, followed by backward elimination of the fixed part.

step(SQ_full_model)

| ##  ## | Backward reduced | | random-effect table: | | |  | | | | | | |
| --- | --- | --- | --- | --- | --- | --- | --- | --- | --- | --- | --- | --- |
| ##  ## | <none> | | Eliminated | | | npar  20 | logLik  -577.14 | AIC  1194.3 | LRT | Df | Pr(>Chisq) | |
| ## | Week in (1 + Week | | \| Group) 1 | | | 18 | -577.66 | 1191.3 | 1.0350 | 2 | 0.59602 | |
| ## | (1 \| Group) | | 0 | | | 17 | -581.17 | 1196.3 | 7.0157 | 1 | 0.00808 | |
| ##  ## | <none> | |  | | |  |  |  |  |  |  | |
| ## | Week in (1 + Week | | \| Group) | | |  |  |  |  |  |  | |
| ## ## | (1 \| Group) **  --- | | | | | |  | |  | | | |
| ##  ## | Signif. codes: 0 '***' 0.001 '**' 0.01 '*' | | | | | | 0.05 '.' 0.1 | | ' ' 1 | | | |
| ##  ## | Backward reduced fixed-effect table:  Degrees of freedom method: Satterthwaite | | | | | |  | |  | | | |
| ## |  | | | | | |  | |  | | | |
| ## |  | Eliminated | | Sum Sq | Mean Sq | | NumDF | DenDF | F value | Pr(>F) | |  |
| ## | RBC | 1 | | 4533 | 4533 | | 1 | 72.356 | 0.0112 | 0.91596 | |  |
| ## | Haematocrit | 2 | | 37429 | 37429 | | 1 | 72.994 | 0.0926 | 0.76175 | |  |
| ## | Prealbumin | 3 | | 132955 | 132955 | | 1 | 71.326 | 0.3278 | 0.56874 | |  |
| ## | Creatinine | 4 | | 77947 | 77947 | | 1 | 70.893 | 0.1916 | 0.66293 | |  |
| ## | Chloride | 5 | | 134358 | 134358 | | 1 | 71.321 | 0.3295 | 0.56777 | |  |
| ## | Urea | 6 | | 203181 | 203181 | | 1 | 70.975 | 0.4966 | 0.48328 | |  |
| ## | Haemoglobin | 7 | | 532098 | 532098 | | 1 | 70.899 | 1.2926 | 0.25939 | |  |
| ## | RDW | 8 | | 710754 | 710754 | | 1 | 71.006 | 1.6969 | 0.19691 | |  |
| ## | Albumin | 9 | | 537646 | 537646 | | 1 | 70.983 | 1.2516 | 0.26703 | |  |
| ## | Bicarbonate | 10 | | 686133 | 686133 | | 1 | 70.887 | 1.5716 | 0.21409 | |  |
| ## | TotalBilirubin | 11 | | 1105630 | 1105630 | | 1 | 72.685 | 2.4762 | 0.11993 | |  |
| ## | Lymphocytes | 12 | | 1553788 | 1553788 | | 1 | 72.679 | 3.3371 | 0.07184 | | . |
| ## | CRP | 13 | | 1410796 | 1410796 | | 1 | 71.003 | 2.8700 | 0.09463 | | . |
| ## | Trp | 0 | | 13221430 | 13221430 | | 1 | 71.473 | 25.8273 | 2.875e-06 | | *** |
| ## | GGT | 0 | | 15055352 | 15055352 | | 1 | 71.506 | 29.4097 | 7.558e-07 | | *** |
| ## | --- |  | |  |  | |  |  |  |  | |  |

## Signif. codes: 0 '***' 0.001 '**' 0.01 '*' 0.05 '.' 0.1 ' ' 1 ##

## Model found:

## SQ ~ Trp + GGT + (1 | Group)

The reduced model has only two factors: tryptophan and GGT. We will refit the reduced model.

formula <- paste(" SQ ~ Trp + GGT + (1 | Group)")

SQ_reduced_model <- lmer(formula, REML = F, data = SQfull) sumSQ_reduced_model <- summary(SQ_reduced_model) sumSQ_reduced_model

| ## | Linear mixed model fit by maximum likelihood . t-tests use Satterthwaite's | | | | | | |
| --- | --- | --- | --- | --- | --- | --- | --- |
| ## | method [lmerModLmerTest] | | | | | | |
| ## | Formula: formula | | | | | | |
| ## | Data: SQfull | | | | | | |
| ##  ## | AIC BIC logLik -2*log(L) df.resid | | | | | | |
| ##  ## | 1180.7 1192.2 -585.4 1170.7 68 | | | | | | |
| ## | Scaled residuals: | | | | | | |
| ## | Min 1Q Median 3Q Max | | | | | | |
| ## | -2.9783 -0.6103 0.1435 0.6328 2.0373 | | | | | | |
| ##  ## | Random effects: | | | | | | |
| ## | Groups Name Variance Std.Dev. | | | | | | |
| ## | Group (Intercept) 86266 293.7 | | | | | | |
| ## | Residual 511917 715.5 | | | | | | |
| ## ## | Number of obs: 73, groups: Group, | 2 |  |  |  |  |  |
| ##  ## | Fixed effects:  Estimate Std. Error |  | df | t | value | Pr(>\|t\|) |  |
| ## | (Intercept) 15455.780 2519.948 |  | 72.367 |  | 6.133 | 4.12e-08 | *** |
| ## | Trp 6.352 1.250 |  | 71.473 |  | 5.082 | 2.87e-06 | *** |
| ##  ## | GGT -10450.604 1927.062  --- |  | 71.506 |  | -5.423 | 7.56e-07 | *** |

#### Notes :

## Signif. codes: 0 '***' 0.001 '**' 0.01 '*' 0.05 '.' 0.1 ' ' 1 ##

## Correlation of Fixed Effects:

## (Intr) Trp ## Trp -0.295

## GGT -0.992 0.206

## fit warnings:

## Some predictor variables are on very different scales: consider rescaling

Stepwise reduction of the full model above yielded a simplified model with only two independent predictors for the SQ score between groups after eliminating the effect of weeks for random slope: tryptophan (fixed effect coefficient = 6.35, *p* < 0.001) and GGT (fixed effect coefficient = -10450.60, *p* < 0.001).

### Visualisation of the linear models

Here, comparing the RBAC and placebo groups, we offer a two-dimensional visual depiction of how tryptophan and GGT could predict SQ.

xlabel <- paste0("GGT (BoxCox \u03BB = ", round(Lamda$GGT,2), ")") ylabel <- paste("SQ (BoxCox \u03BB = ", round(Lamda$SQ,2), ")")

plotGGT <- SQfull %>%

ggplot(aes(y = SQ, x = GGT , col = Group)) + geom_point() +

geom_smooth(method = 'lm', se = F) + xlab(xlabel) +

ylab(ylabel)

xlabel <- paste0("Trp (BoxCox \u03BB = ", round(Lamda$Trp,2), ")") plotTrp <- SQfull %>%

ggplot(aes(y = SQ, x = Trp, col = Group)) +

geom_point() +

geom_smooth(method = 'lm', se = F) + xlab(xlabel) +

ylab(ylabel)

figure2 <- ggarrange(plotTrp, plotGGT,

labels = c("(I)", "(II)"),

ncol = 2, nrow = 1, common.legend = TRUE)

figure2


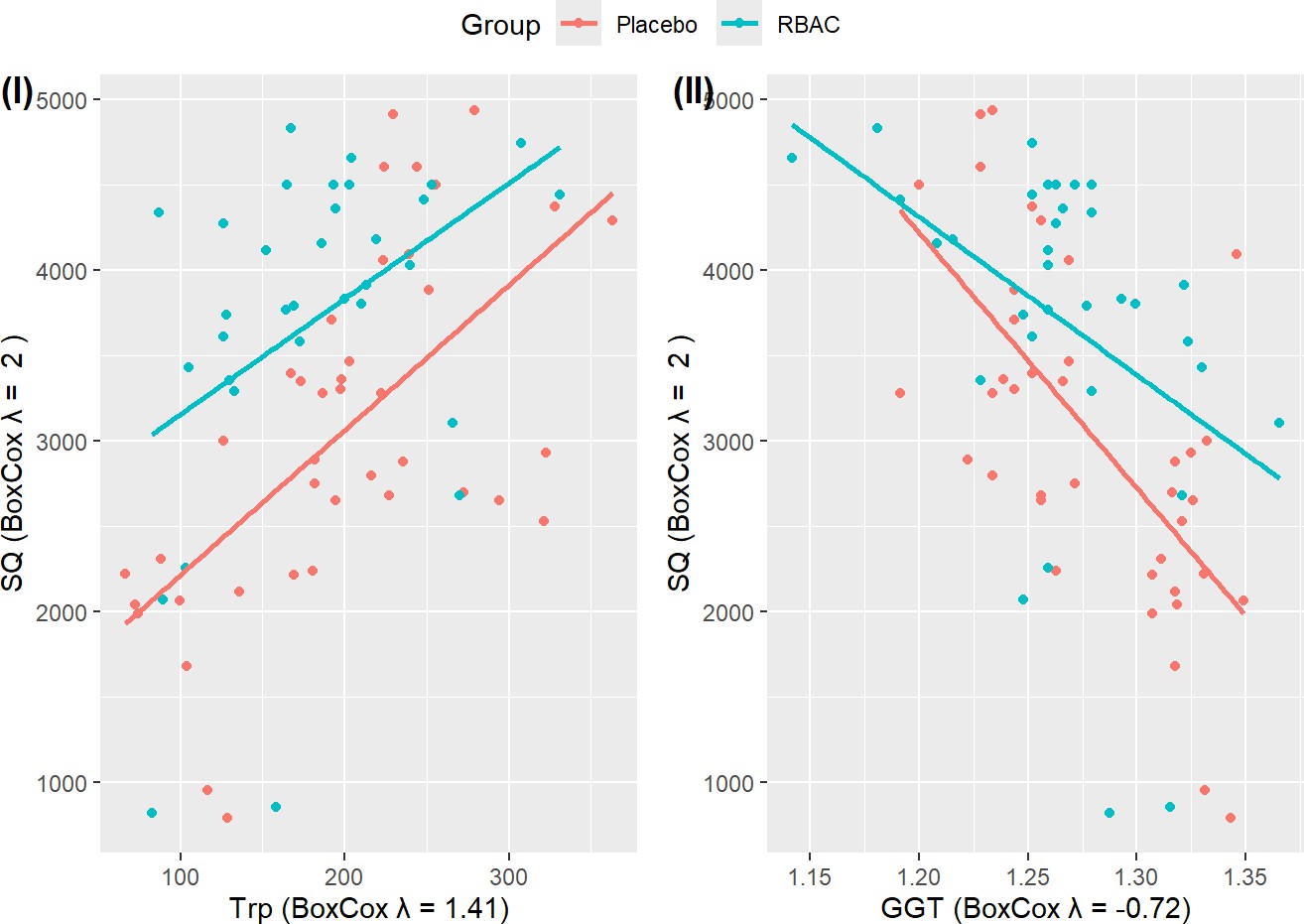


#### Notes :

The following observations can be made:

1. The increase in tryptophan is accompanied by the rise in SQ for both groups at a similar rate. However, the RBAC group had a consistently higher SQ than the placebo group at any level of tryptophan. Hence, there appeared to be an additive effect between RBAC and tryptophan on SQ.
2. Higher GGT predicted lower SQ, with both the RBAC and placebo groups showing similar levels of SQ at low GGT levels. However, the rate of deterioration between groups differed with increasing GGT, with the drop in SQ being much steeper in the placebo group.

## Conclusion

Among all the blood markers available for analysis, tryptophan is one of the two most prominent independent factors that could predict SQ, with the other being GGT, a liver function marker.

1. Charles Sturt University, [sooi@csu.edu.au](mailto:sooi@csu.edu.au) [(mailto:sooi@csu.edu.au)](mailto:sooi@csu.edu.au)↩
2. University of Sydney, [benjamin.kimble@sydney.edu.au](mailto:benjamin.kimble@sydney.edu.au) [(mailto:benjamin.kimble@sydney.edu.au)](mailto:benjamin.kimble@sydney.edu.au)↩
3. Charles Sturt University, [benjamin.pak@unsw.edu.au](mailto:benjamin.pak@unsw.edu.au) [(mailto:benjamin.pak@unsw.edu.au)](mailto:benjamin.pak@unsw.edu.au)↩
4. Charles Sturt University, [pmicalos@csu.edu.au](mailto:pmicalos@csu.edu.au) [(mailto:pmicalos@csu.edu.au)](mailto:pmicalos@csu.edu.au)↩
5. Charles Sturt University, [spak@csu.edu.au](mailto:spak@csu.edu.au) [(mailto:spak@csu.edu.au)](mailto:spak@csu.edu.au)↩
